# Homoserine Lactone as a Structural Key Element for the Synthesis of Multifunctional Polymers

**DOI:** 10.3390/polym9040130

**Published:** 2017-04-05

**Authors:** Fabian Marquardt, Stefan Mommer, Justin Lange, Pascal M. Jeschenko, Helmut Keul, Martin Möller

**Affiliations:** Institute of Technical and Macromolecular Chemistry, RWTH Aachen University and DWI-Leibniz-Institute for Interactive Materials, Forckenbeckstr. 50, D-52056 Aachen, Germany; Marquardt@dwi.rwth-aachen.de (F.M.); Mommer@dwi.rwth-aachen.de (S.M.); Justin.Lange@rwth-aachen.de (J.L.); Pascal.Jeschenko@rwth-aachen.de (P.M.J.)

**Keywords:** homoserine lactone, thiolactone-lactone coupler, functional polyamide, functional polyether, polyaddition reaction

## Abstract

The use of bio-based building blocks for polymer synthesis represents a milestone on the way to “green” materials. In this work, two synthetic strategies for the preparation of multifunctional polymers are presented in which the key element is the functionality of homoserine lactone. First, the synthesis of a bis cyclic coupler based on a thiolactone and homoserine lactone is displayed. This coupler was evaluated regarding its regioselectivity upon reaction with amines and used in the preparation of multifunctional polymeric building blocks by reaction with diamines. Furthermore, a linear polyglycidol was functionalized with homoserine lactone. The resulting polyethers with lactone groups in the side chain were converted to cationic polymers by reaction with 3-(dimethylamino)-1-propylamine followed by quaternization with methyl iodide.

## 1. Introduction

**Scheme 1:**
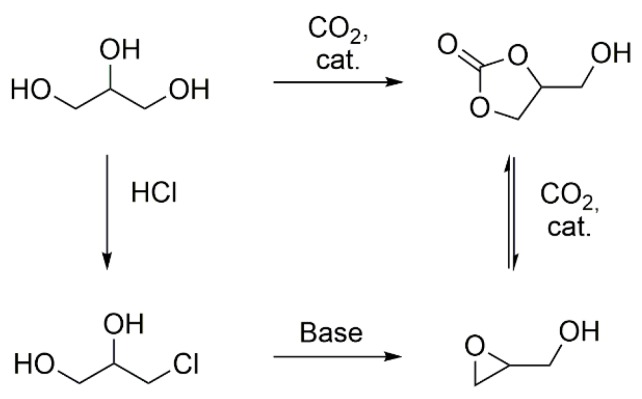
Strategies for the synthesis of glycerol carbonate and glycidol from glycerol [9].

In recent years, the increasing shortage of resources and the consequential sustainable awareness has led to a change in mindset of chemists towards bio-based and renewable resources. The development of enzymatic polymer synthesis [1], multifunctional high performance fibers [2], membranes for water purification [3], transparent substrates for use in electronics from wood [4,5] , amino-acid based ionic liquids [6], and asymmetric building blocks [7] are results of this rethinking process. Another approach towards sustainability involves the utilization of bio-based by-products. Glycerol is a readily available, major by-product in the production of biodiesel, and thus used as a starting material for the synthesis of monomers, such as epichlorohydrin and glycidol [8], or building blocks like glycerol carbonate (Scheme 1) [9].

Glycidol acts as the monomer in the synthesis of linear, branched and star-like polyglycidols [10,11,12]. Polyglycidol is soluble in aqueous media, shows no toxicity towards cells and is licensed by the Food and Drug Administration (FDA) [13,14]. It is a highly functionalized polymer with a hydroxyl group in every repeating unit, allowing for various further modifications [15,16]. Hans et al. presented the application of polyglycidol, as a multifunctional macroinitiator, for the ring-opening polymerization of ɛ-caprolactone [17,18]. The reaction was carried out by chemical and enzymatic catalysis, leading to densely and loosely grafted polyglycidols, respectively. The loosely grafted polymers showed enhanced hydrolytic degradation of the poly(caprolactone) side chains allocated to the free hydroxyl groups of the polyglycidol backbone.

The hydrolytic degradation of poly(caprolactone) was further increased by functionalization of the polyglycidol macroinitiator with phosphonate/phosphonic acid groups [19,20]. Polyglycidol was functionalized in a Michael-type addition with diethylvinyl phosphonate and subsequent dealkylation of the pendant phosphonate groups [21]. Additionally, multifunctional polyglycidols carrying phosphonic acid and acrylate moieties were examined as UV-active adhesion promoters for a hydrogel coating on stainless steel wires [22].

Recently, we developed a synthetic strategy for the post-polymerization functionalization of polyglycidol with pendant phosphate groups by reaction with diethyl chlorophosphate [23]. The diethyl phosphate moieties were subsequently (mono-)dealkylated. This method allowed for the tailoring of the concentration of pendant phosphate/phosphoric acid groups introduced into the polyglycidol.

**Scheme 2:**
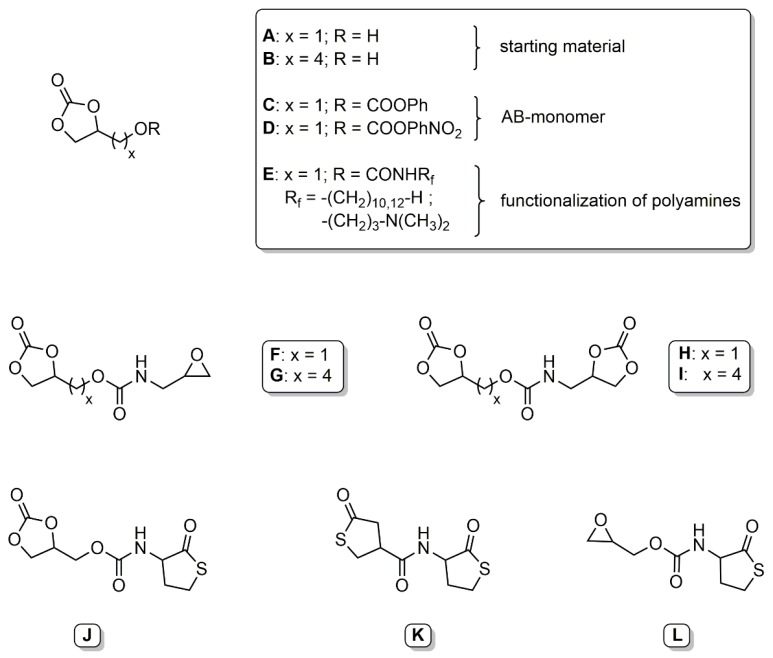
Overview of presented AB-monomers/couplers.

Glycerol carbonate has been used as starting material in the synthesis of various bifunctional couplers. Ubaghs et al. developed a (2-oxo-1,3-dioxolan-4-yl)methyl phenyl carbonate (Scheme 2, **C**) as an AB-type monomer for the isocyanate-free synthesis of poly(urethane)s with pendant hydroxyl groups [24]. This carbonate was used later by Pasquier et al. as a coupling agent to link functional poly- or monodisperse building blocks [25]. These couplers are selectively reacted with an amine at the phenyl carbonate moiety at room temperature, followed by ring-opening at the ethylene carbonate at 60 °C. The glycerol carbonate was functionalized with various cationic and hydrophobic amines and linked to a hyperbranched poly(ethylene imine) to determine the antimicrobial properties in aqueous solution and as surface coatings (Scheme 2, **E**) [25,26]. Fricke et al. further developed this concept by synthesizing more reactive 4-nitrophenyl-carbonate couplers (Scheme 2, **D**). These couplers were used to introduce a cyclic carbonate moiety to various amino acid derivatives and glucosamine [27].

He et al. presented two new types of bifunctional couplers based on glycerol carbonate [28]. The first series of couplers carried a five-membered cyclic carbonate and an epoxy group (Scheme 2, **F** and **G**). These coupling agents showed a low selectivity towards primary amines and a very high selectivity towards secondary amines. Introduction of an alkyl chain as a spacer shifted the selectivity towards the epoxide. The second series of couplers carried two five-membered cyclic carbonate rings (Scheme 2, **H** and **I**). The carbonate groups showed different reactivity based on the distance of the carbonate from the linking functional group.

Recently, we presented various homocysteine thiolactone-functional couplers. The homocysteine thiolactone moiety has been thoroughly investigated in regard to its reactivity with amines and subsequent thiol-ene reactions [29,30]. A bifunctional coupler with an ethylene carbonate and a thiolactone functionality was synthesized starting from either (2-oxo-1,3-dioxolan-4-yl)methyl phenyl carbonate or glycerol chloroformate (Scheme 2, **J**) [31,32]. Both functional groups were selectively addressed by an amine, dependent on the temperature, allowing the addition of two different amines. The released thiol was further reacted with methyl acrylate in a Michael-type addition to give the corresponding thioether. The released hydroxyl groups were addressed with an acyl halide. Additionally, a (bis)thiolactone coupler was prepared by reaction of γ-thiobutyrolactone β-acid chloride with homocysteine thiolactone (Scheme 2, **K**) [33]. The (bis)thiolactone showed no regioselective ring-opening of the thiolactone under the specific reaction conditions. Reaction with two different diamines gave thiol-functional polyamides. A novel epoxy thiolactone coupler was presented as a versatile tool for polymeric materials (Scheme 2, **L**) [34]. The coupler was used in multicomponent reactions to link up to four different buildings blocks and was converted with amines to poly(thioether urethane)s. Functional gels were generated by either thiol-epoxy polymerization under basic conditions or multicomponent reaction with a triacrylate.

Herein we present two different strategies for the introduction of homoserine lactone into polymeric materials: (i) Synthesis of a thiolactone-lactone coupler; determination of the selectivity of both rings towards amines and use of the coupler for the preparation of polymeric building blocks; (ii) Functionalization of polyglycidol with homoserine lactone; ring-opening of the lactone with an amine and investigation of further modification possibilities.

## 2. Materials and Methods

### 2.1. Materials

Thioacetic acid (96%, Sigma Aldrich Chemie GmbH, Steinheim, Germany), itaconic acid (99%, Alfa Aesar, Karlsruhe, Germany), thionyl chloride (99.7%, Thermo Fisher Acros Organics, Geel, Belgium), hexylamine (99%, Sigma Aldrich Chemie GmbH, Steinheim, Germany), methyl acrylate (99%, Sigma Aldrich Chemie GmbH, Steinheim, Germany), poly(ethylene glycol) diamine (*M* = 368.47 g·mol^−1^, Iris Biotech GmbH, Marktredwitz, Germany), potassium *tert*-butoxide (1 M solution in THF, Sigma Aldrich Chemie GmbH, Steinheim, Germany), diglyme (≥99%, extra dry, over molecular sieves, Thermo Fisher Acros Organics, Geel, Belgium), pyridine (99.5%, extra dry, over molecular sieves, Thermo Fisher Acros Organics, Geel, Belgium), 4-nitrophenyl chloroformate (>98%, TCI), 3-(dimethylamino)-1-propylamine (99%, Thermo Fisher Acros Organics, Geel, Belgium), DL-homoserine lactone hydrobromide (99%, Sigma Aldrich Chemie GmbH, Steinheim, Germany), 4-dimethylaminopyridine (>98%, Sigma Aldrich Chemie GmbH, Steinheim, Germany), triethylamine (≥99.5%, anhydrous, Sigma Aldrich Chemie GmbH, Steinheim, Germany), methyl iodide (>99%, Sigma Aldrich Chemie GmbH, Steinheim, Germany), tetrahydrofuran (99.8%, stabilizer free, extra dry, Thermo Fisher Acros Organics, Geel, Belgium), chloroform (99.9%, extra dry, over molecular sieves, Thermo Fisher Acros Organics, Geel, Belgium), *N*,*N*-dimethylformamide (99.8%, extra dry, over molecular sieves, Thermo Fisher Acros Organics, Geel, Belgium), methanol (≥99.8%, p.a., Th. Geyer CHEMSOLUTE^®^, Renningen, Germany) and dichloromethane (≥99.8%, anhydrous, Sigma Aldrich Chemie GmbH, Steinheim, Germany) were used as received.

3-Phenyl-1-propanol (99%, Sigma Aldrich Chemie GmbH, Steinheim, Germany) was stirred with calcium hydride for 24 h and then distilled. Ethoxyethyl glycidyl ether (EEGE) was synthesized from 2,3-epoxypropan-1-ol (glycidol) and ethyl vinyl ether according to Fitton et al. [35], purified by distillation, and stored under a nitrogen atmosphere over a molecular sieve (3 Å).

Water-sensitive reactions were carried out in a nitrogen atmosphere. Nitrogen (Linde 5.0) was passed over a molecular sieve (4 Å) and finely distributed potassium on aluminum oxide.

### 2.2. Measurements

^1^H NMR and ^13^C NMR spectra were recorded on a Bruker DPX-400 FT-NMR spectrometer (Bruker Corporation, Billerica, MA, USA) at 400 and 101 MHz, respectively. Deuterated dimethyl sulfoxide (DMSO-*d*_6_), deuterated dimethylformamide (DMF-*d*_7_) and deuterium oxide (D_2_O) were used as solvents. The residual solvent signal was used as the internal standard. Coupling constants ***J_xy_*** are given in Hz.

Molecular weights (*M*_n,SEC_) and molecular weight distributions (*Đ*) were determined by size exclusion chromatography (SEC). SEC analyses were carried out with DMF (HPLC grade, VWR) as the eluent. SEC was performed using an Agilent 1100 system (Agilent Technologies, Santa Clara, CA, USA) equipped with a dual RI-/Visco detector (ETA-2020, WGE Dr. Bures GmbH & Co KG, Dallgow-Doeberitz, Germany). The eluent contained 1 g·L^−1^ LiBr (≥99%, Aldrich). The sample solvent contained traces of distilled water as the internal standard. One pre-column (8 mm × 50 mm) and four GRAM gel columns (8 mm × 300 mm, Polymer Standards Service) were applied at a flow rate of 1.0 mL·min^−1^ at 40 °C. The diameter of the gel particles measured 10 µm, the nominal pore widths were 30, 100, 1000 and 3000 Å. Calibration was achieved using narrowly distributed poly(methyl methacrylate) standards (Polymer Standards Service). Results were evaluated using the PSS WinGPC UniChrom software (Version 8.1.1, PSS Polymer Standards Service GmbH, Mainz, Germany).

FTIR spectra were recorded on a Thermo Nicolet Nexus 470 FTIR spectrometer at 25 °C. The samples were recorded between KBr disks and scanned over a range of 400–4000 cm^−1^.

ESI mass spectra were recorded on a Finnigan SSQ 7000 spectrometer (Thermo Scientific (Finnigan Corporation), Waltham, MA, USA) and HRMS spectra on a Thermo Scientific LTQ Orbitrap XL spectrometer (Thermo Scientific, Waltham, MA, USA).

NALDI-TOF mass spectrometry was performed on a Bruker ultrafleXtreme (Bruker Corporation, Billerica, MA, USA) equipped with a 337 nm smartbeam laser in the reflective mode. Tetrahydrofuran (THF) solutions of analyte (20 μL of 10 mg·mL^−1^) were prepared, 2 μL thereof were applied on the sample plate. Laser shots (6000) with 24% up to 60% laser power were collected. The laser repetition rate was 1000 Hz.

Elemental analysis was performed on an Elementar Vario EL (Elementar Analysensysteme GmbH, Langenselbold, Germany). Results are reported in weight percent.

Dialysis was performed in methanol using Biotech CE Tubing (MWCO: 100–500 D, 3.1 mL·cm^−1^, Spectrum Laboratories, Inc., Rancho Dominguez, CA, USA). The membrane was washed for 15 min in water before use to remove the sodium azide solution.

### 2.3. Synthesis of the Thiolactone-Homoserine Lactone Coupler (**1**)

To an ice-cold mixture of γ-thiobutyrolactone β-acid chloride (1.788 g, 10.86 mmol) and DL-homoserine lactone hydrobromide (2.000 g, 10.86 mmol), pyridine (8.74 mL) was slowly added and stirred for 16 h at room temperature. Excess pyridine was removed under reduced pressure. The crude product was purified by flash column chromatography on silica gel using DCM/MeOH (95:5) as the eluent. The coupler **1** was obtained as a slightly yellow solid (1.80 g, 82%). *R*_f_ = 0.15, *T*_m_ = 157.0 °C. ^1^H NMR (400 MHz, DMSO-*d*_6_): δ = 2.10–2.22 (m, 1H, NCH*CH*_2_), 2.38–2.46 (m, 1H, NCH*CH*_2_), 2.65–2.84 (m, 2H, SC=O*CH*_2_), 3.31–3.40 (m, 1H, SCH_2_*CH*), 3.41–3.51 (m, 1H, S*CH*_2_CH), 3.57–3.67 (m, 1H, S*CH*_2_CH), 4.17–4.26 (m, 1H, O*CH*_2_), 4.31–4.39 (m, 1H, O*CH*_2_), 4.54–4.64 (m, 1H, N*CH*), 8.68 (d, *J* = 7.8 Hz, 1H, NH) ppm. ^13^C NMR (100 MHz, DMSO-*d*_6_): δ = 28.1 (NCH*CH*_2_), 34.8 (S*CH*_2_), 42.8 (S*CH*_2_CH), 43.5 (SC=O*CH*_2_), 48.2 (N*CH*), 65.3 (O*CH*_2_), 170.7 (N*C=O*), 175.0 (*OC=O*), 206.5 (S*C=*O) ppm. IR (KBr): υ_max_ = 3371 (m), 3312 (s), 3083 (w), 2929 (w), 1790 (m), 1771 (s), 1700 (s), 1652 (vs), 1551 (s), 1372 (w), 1284 (w) 1161 (m), 1022 (m), 985 (w), 654 (w). *m/z* for C_9_H_11_NO_4_S: measured: (*M* + Cl)^−^ 264.017/269.012, (*M* + Cl)^−^ calculated: 264.01. Elemental analysis: C_9_H_11_NO_4_S (*M* = 229.25 g·mol^−1^), measured: C: 46.83, H: 4.82, N: 5.73, calculated: C: 47.15, H: 4.84, N: 6.11.

### 2.4. Conversion of Homoserine Lactone with Hexylamine in a 1:1 Molar Ratio

Hexylamine (115 μL, 0.877 mmol) was added to a stirred solution of the coupler **1** (201 mg, 0.877 mmol) in dry DMF (1.75 mL, 0.5 M) at 0 °C. The reaction mixture was allowed to warm slowly to room temperature and stirred at room temperature for 16 h. The solvent was evaporated under reduced pressure to give the crude product. Purification by flash column chromatography on silica gel using DCM/MeOH (95:5) as the eluent gave the addition product **2** (240 mg, 83%) as a waxy solid. *R*_f_ = 0.19. ^1^H NMR (400 MHz, DMSO-*d*_6_): δ = 0.85 (t, *J* = 6.3 Hz, 3H, *CH*_3_CH_2_CH_2_), 1.18–1.30 (m, 6H, CH_3_*CH*_2_*CH*_2_*CH*_2_CH_2_), 1.31–1.41 (m, 2H, *CH*_2_CH_2_NH), 2.06–2.44 (m, 4H, *CH*_2_CHCH_2_SH, NHCH*CH*_2_), 2.53–2.65 (m, 2H, CH_2_CH*CH*_2_SH) 2.75–2.85 (m, 1H, CH_2_*CH*CH_2_SH), 2.95–3.09 (m, 2H, CH_2_*CH*_2_NH), 4.14–4.25 (m, 1H, NHCHCH_2_*CH*_2_), 4.28–4.40 (m, 1H, NHCHCH_2_*CH*_2_), 4.41–4.62 (m, 1H, NH*CH*CH_2_), 7.79–7.91 (m, 1H, NH), 8.44–8.61 (m, 1H, NH) ppm. ^13^C NMR (100 MHz, DMSO-*d*_6_): δ = 13.9 (*CH*_3_CH_2_CH_2_), 22.1 (CH_2_CH*CH*_2_SH), 25.9 (CH_3_*CH*_2_CH_2_), 26.1 (CH_3_CH_2_CH_2_*CH*_2_CH_2_), 28.1 (CH_3_CH_2_CH_2_CH_2_*CH*_2_), 29.0 (NHCH*CH*_2_), 31.0 (CH_3_CH_2_*CH*_2_CH_2_), 37.0 (CH_2_*CH*_2_NH), 38.6 (*CH*_2_CHCH_2_SH), 45.1 (CH_2_*CH*CH_2_SH), 54.9 (NH*CH*CH_2_), 65.3 (NHCHCH_2_*CH*_2_), 169.7 (NH*C=O*), 172.7 (*C=O*NH), 175.3 (*OC=O*) ppm. *m/z* for C_15_H_26_N_2_O_4_S: measured: (*M* + Na)^+^ 353.128, calculated: (*M* + Na)^+^ 353.15.

### 2.5. Conversion of Homoserine Lactone with Hexylamine in a 1:2 Molar Ratio

Hexylamine (235 μL, 1.79 mmol) was added to a stirred solution of the coupler **1** (205 mg, 0.894 mmol) in dry DMF (1.79 mL, 0.5 M) at 40 °C. The reaction mixture was stirred at 40 °C for 20 h. The solvent was evaporated under reduced pressure to give the crude product. Purification by flash column chromatography on silica gel using DCM/MeOH (95:5) as the eluent gave the addition product **3** (159 mg, 34%) as a waxy solid. *R*_f_ = 0.24. ^1^H NMR (400 MHz, DMSO-*d*_6_): δ = 0.85 (t, *J* = 6.8 Hz, 6H, *CH*_3_CH_2_CH_2_), 1.14–1.30 (m, 12H, CH_3_*CH*_2_*CH*_2_*CH*_2_CH_2_), 1.31–1.47 (m, 4H, *CH*_2_CH_2_NH), 1.54–1.72 (m, CH*CH*_2_CH_2_OH), 1.78–2.05 (m, 1H, CH*CH*_2_CH_2_OH), 2.16 (t, *J* = 8.0 Hz, 1H, SH) 2.23–2.44 (m, 2H, *CH*_2_CHCH_2_SH), 2.52–2.63 (m, 2H, CH_2_CH*CH*_2_SH), 2.76–2.86 (m, 1H, CH_2_*CH*CH_2_SH), 2.92–3.14 (m, 4H, CH_2_*CH*_2_NH), 3.37–3.52 (m, 2H, CHCH_2_*CH*_2_OH), 4.14–4.24 (m, 1H, *CH*CH_2_CH_2_OH), 4.38–4.49 (m, 1H, OH), 7.79–8.08 (m, 3H, NH) ppm. ^13^C NMR (100 MHz, DMSO-*d*_6_): δ = 13.9 (*CH*_3_CH_2_CH_2_), 22.0 (CH_2_CH*CH*_2_SH), 25.9 (CH_3_*CH*_2_CH_2_), 29.0 (CH_3_CH_2_CH_2_CH_2_*CH*_2_), 30.9 (CH_3_CH_2_*CH*_2_CH_2_), 34.3 (CH_3_CH_2_CH_2_*CH*_2_CH_2_), 34.8 (CH_2_*CH*_2_NH), 36.9 (CH*CH*_2_CH_2_OH), 38.5 (*CH*_2_CHCH_2_SH), 50.1 (CH_2_*CH*CH_2_SH), 54.6 (*CH*CH_2_CH_2_OH), 57.8 (CHCH_2_*CH*_2_OH), 171.5 (CH*C=O*NHCH_2_), 172.5 (NH*C=O*CH_2_), 173.0 (CH*C=O*NHCH) ppm. *m*/*z* for C_21_H_45_N_3_O_4_S: measured: (*M* + Na)^+^ 454.268, calculated: (*M* + Na)^+^ 454.27.

### 2.6. General Procedure for the Polyaddition of PEG-Diamine to Homoserine Lactone in the Presence of Methyl Acrylate

Methyl acrylate (1 eq.) and PEG-diamine (1 eq.) were added to a solution of coupler **1** (1 eq.) in DMF. The solution was stirred for 16 h at 90 °C. The solvent was removed under reduced pressure and the crude product was obtained as a yellow oil. **4a**–**c** were synthesized using DMF solutions of different concentrations. **4d** was synthesized in bulk. Detailed information can be found in the supporting information (Table S3). As the polyaddition procedure does not require any purification of the product, the product can be considered as obtained in quantitative yield.

^1^H NMR (400 MHz, DMSO-*d*_6_): δ = 1.54–1.91 (m, NHCH*CH*_2_CH_2_OH), 2.06–2.75 (m, *CH*_2_CH*CH*_2_SCH_2_*CH*_2_), 2.97–3.10 (m, CH_2_CHCH_2_S*CH*_2_CH_2_), 3.12–3.27 (m, CH_2_*CH*CH_2_SCH_2_CH_2_), 3.28–3.43 (m, C=ONH*CH*_2_CH_2_OCH_2_), 3.44–3.57 (m, NHCH_2_*CH*_2_O*CH*_2_*CH*_2_O*CH*_2_), 3.58–3.72 (m, NHCHCH_2_*CH*_2_OH, SCH_2_CH_2_C=OO*CH*_3_), 4.12–4.57 (m, NH*CH*CH_2_CH_2_*OH*), 7.66–8.67 (m, NH) ppm.

### 2.7. Synthesis of Poly(Glycidyl-4-Nitrophenylcarbonate)_26_ (P(G^NPC^)_26_) (**6**)

PG_26_ (**5**) (0.990 g, 13.36 mmol OH) was dissolved in pyridine (9.28 mL). 4-Nitrophenyl chloroformate (2.963 g, 14.70 mmol) was dissolved in DCM (9.0 mL) and added to the polymer solution via syringe pump in 30 min at 0 °C. The solution was stirred for 20 h at room temperature. The crude product was washed with water (30 mL), 1 M HCl (aq.) (3 × 30 mL) and saturated NaCl solution (aq., 30 mL). The organic phase was separated, dried over Na_2_SO_4_ and precipitated in cold MeOH. The solvent was removed under reduced pressure and polymer **6** was obtained as a colorless solid (2.922 g, 91%). ^1^H-NMR (400 MHz, DMSO-*d*_6_): δ = 1.69–1.83 (m, ArCH_2_*CH*_2_), 2.54–2.61 (m, Ar*CH*_2_CH_2_), 3.68–3.72 (m, ArCH_2_CH_2_*CH*_2_, O*CH*_2_*CH*(CH_2_OC=OOArNO_2_)O), 4,26–4.42 (d, *CH*_2_OC=OOArNO_2_), 7.12–7.21 (m, *Ar*CH_2_CH_2_), 7.41 (s, CH_2_OC=OO*Ar*NO_2_), 8.17 (s, CH_2_OC=OO*Ar*NO_2_) ppm. ^13^C NMR (100 MHz, DMSO-*d*_6_): δ = 30.8 (ArCH_2_*CH*_2_), 31.6 (Ar*CH*_2_), 68.3 (O*CH*_2_CH(CH_2_OC=OOArNO_2_)O), 76.4 (OCH_2_*CH*(CH_2_OC=OOArNO_2_)O), 76.5 (ArCH_2_CH_2_*CH*_2_), 76.6 (OCH_2_CH(*CH*_2_OC=OOArNO_2_)O), 122.3 (CH_2_OC=OO*Ar*NO_2_), 125.2 (CH_2_OC=OO*Ar*NO_2_), 125.7 (*Ar*CH_2_), 128.2 (*Ar*CH_2_), 128.3 (*Ar*CH_2_), 141.6 (*Ar*CH_2_), 145.0 (CH_2_OC=OO*Ar*NO_2_), 152.0 (*OC=OO*), 155.1 (CH_2_OC=OO*Ar*NO_2_) ppm.

### 2.8. Synthesis of Poly(Glycidyl Homoserine Lactonylcarbamate)_26_ (P(G^HSL^)_26_) (**7**)

Polymer **6** (1.000 g, 4.181 mmol OH) was dissolved in DMF (20 mL), and 4-(dimethylamino)pyridine (0.051 g, 0.418 mmol) and DL-homoserine lactone hydrobromide (0.761 g, 4.181 mmol) were added. The mixture was cooled to 0 °C and triethylamine (0.846 g, 8.361 mmol) was added over 1 h via syringe pump. The solution was stirred for 20 h at room temperature. DMF was removed under reduced pressure at 50 °C and the crude product was precipitated in MeOH. Drying under reduced pressure at 50 °C gave polymer **7** as a colorless solid (0.648 g, 77%). ^1^H NMR (400 MHz, DMF-*d*_7_): δ = 1.81–1.90 (m, ArCH_2_*CH*_2_), 2.27–2.45 (m, NHCH*CH*_2_), 2.49–2.63 (m, NHCH*CH*_2_), 2.65–2.71 (m, ArCH_2_*CH*_2_), 3.61–3.86 (m, ArCH_2_CH_2_*CH*_2_, O*CH*_2_*CH*(CH_2_OC=OR)O), 4.09 (s, OCH_2_CH(*CH*_2_OC=OR)O), 4.20–4.37 (m, NH*CH*CH_2_, OCH2CH(*CH*_2_OC=OR)O), 4.37–4.49 (m, NHCHCH_2_*CH*_2_), 4.51–4.66 (m, NHCHCH_2_*CH*_2_), 7.23–7.36 (m, *Ar*CH_2_CH_2_), 7.66 (s, NH) ppm. ^13^C NMR (100 MHz, DMF-*d*_7_): δ = 28.7 (NHCH*CH*_2_), 31.6 (ArCH_2_*CH*_2_), 32.2 (Ar*CH*_2_), 50.3 (NH*CH*CH_2_), 65.7 (NHCHCH_2_*CH*_2_), 64.7, 69.5, 78.0 (ArCH_2_CH_2_*CH*_2_, O*CH*_2_*CH*(*CH*_2_OR)O), 128.7 (*Ar*CH_2_), 128.8 (*Ar*CH_2_), 156.6 (*OC=O*NH), 175.8 (NHCH*C=O*O) ppm.

### 2.9. Synthesis of P(G^HSL,o^)_26_ (**8**)

Polymer **7** (0.263 g, 1.307 mmol HSL) was dissolved in DMF (4 mL), and 3-(dimethylamino)-1-propylamine (0.339 g, 3.268 mmol) and 4-(dimethylamino)pyridine (0.016 g, 0.131 mmol) were added. The mixture was stirred for 20 h at room temperature. DMF was removed under reduced pressure at 50 °C and the crude product was precipitated in diethyl ether. Dialysis in MeOH gave **8** as a slightly yellow solid (0.373 g, 94%).

^1^H NMR (400 MHz, DMSO-*d*_6_): δ = 1.41–1.58 (m, NHCH_2_*CH*_2_CH_2_NMe_2_), 1.66–1.77 (m, ArCH_2_*CH*_2_, NHCH*CH*_2_CH_2_OH), 2.09 (s, N*Me*_2_), 2.18 (m, NHCH_2_CH_2_*CH*_2_NMe_2_), 2.55–2.61 (m, Ar*CH*_2_CH_2_), 3.03–3.10 (m, NH*CH*_2_CH_2_CH_2_NMe_2_), 3.47–3.73 (m, ArCH_2_CH_2_*CH*_2_, O*CH*_2_*CH*(CH_2_OR)O, NHCHCH_2_*CH*_2_OH), 3.81–4.25 (m, OCH_2_CH(*CH*_2_OR)O, NH*CH*CH_2_CH_2_OH), 7.14–7.36 (m, *Ar*CH_2_, NH), 7.81–8.02 (m, NH) ppm. ^13^C NMR (100 MHz, DMSO-*d*_6_): δ = 26.9 (NHCH_2_*CH*_2_CH_2_NMe_2_), 31.0 (ArCH_2_*CH*_2_), 31.7 (Ar*CH*_2_CH_2_), 35.1 (NHCH*CH*_2_CH_2_OH), 37.2 (NH*CH*_2_CH_2_CH_2_NMe_2_), 45.2 (N*Me*_2_), 52.2 (NH*CH*CH_2_CH_2_OH), 56.8 (NHCHCH_2_*CH*_2_OH), 57.7 (NHCH_2_CH_2_*CH*_2_NMe_2_), 63.8, 68.7, 77.8 (ArCH_2_CH_2_*CH*_2_, O*CH*_2_*CH*(*CH*_2_OR)O), 125.8 (*Ar*CH_2_), 128.3 (*Ar*CH_2_), 128.4 (*Ar*CH_2_), 141.8(*Ar*CH_2_), 156.0 (O*C=O*NH), 171.9 (CH*C=O*NH) ppm.

### 2.10. Synthesis of P(G^HSL,oq^)_26_ (**9**)

Polymer **8** (0.250 g, 0.824 mmol –NMe_2_ groups) was dissolved in MeOH (5 mL) and methyl iodide (0.175 g, 1.236 mmol) was added. The solution was stirred under reflux for 20 h. Removal of MeOH and excess methyl iodide under reduced pressure at 50 °C gave polymer **9** as a yellow solid (0.324 g, 88%). ^1^H NMR (400 MHz, D_2_O): δ = 1.95–2.08 (m, ArCH_2_*CH*_2_, NHCH_2_*CH*_2_CH_2_NMe_3_, NHCH*CH*_2_CH_2_OH), 2.66–2.74 (m, Ar*CH*_2_CH_2_), 3.18 (s, N*Me_3_*), 3.25–3.47 (m, NH*CH*_2_CH_2_*CH*_2_NMe_3_), 3.72–3.87 (m, ArCH_2_CH_2_*CH*_2_, O*CH*_2_*CH*(CH_2_OR)O, NHCHCH_2_*CH*_2_OH), 4.12–4.37 (m, OCH_2_CH(*CH*_2_OR)O, NH*CH*CH_2_CH_2_OH), 7.33–7.42 (m, *Ar*CH_2_) ppm. ^13^C NMR (100 MHz, D_2_O): δ = 22.8 (NHCH_2_*CH*_2_CH_2_NMe_3_), 33.8 (NHCH*CH*_2_CH_2_OH), 36.2 (NH*CH*_2_CH_2_CH_2_NMe_3_), 52.8 (NH*CH*CH_2_CH_2_OH), 53.2 (N*Me*_3_), 58.0 (NHCHCH_2_*CH*_2_OH), 64.2 (NHCH_2_CH_2_*CH*_2_NMe_3_), 64.5 (OCH_2_CH(*CH*_2_OR)O), 68.8, 76.6, 77.3 (ArCH_2_CH_2_*CH*_2_, O*CH*_2_*CH*(CH_2_OR)O), 128.2 (*Ar*CH_2_), 128.8 (*Ar*CH_2_), 141.0 (*Ar*CH_2_), 157.5 (O*C=O*NH), 174.7 (CH*C=O*NH) ppm.

## 3. Results and Discussion

In the current work, we present the synthesis of a novel bis cyclic compound which is used as an AA-type monomer or as a coupling agent. The structural key element is homoserine lactone derived from the bio-based building block homoserine. The bis cyclic thiolactone-homoserine lactone monomer is examined in regard to the reactivity of each heterocycle upon amine addition and polymerization with a diamine. Furthermore, polyglycidol is functionalized with homoserine lactone to prepare multifunctional polyethers.

### 3.1. Synthesis of the Thiolactone-Homoserine Lactone Coupler (**1**)

**Scheme 3:**
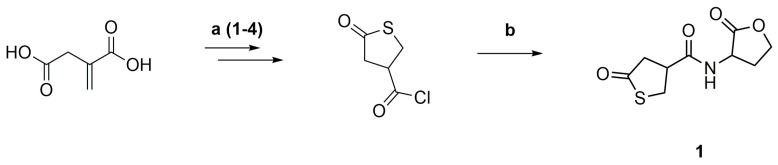
Synthesis of the thiolactone-lactone coupler (**1**) from itaconic acid and thioacetic acid (**1.** THF, reflux, 4h; **2.** HCl, reflux, 6 h; **3.** TFA, reflux, 1 h; **4.** SOCl_2_, CHCl_3_, reflux, 6 h) (**a**), followed by reaction with DL-homoserine lactone hydrobromide and pyridine (rt, 16 h) (**b**).

γ-Thiobutyrolactone β-acid chloride was synthesized from itaconic acid and thioacetic acid, followed by reaction with thionyl chloride and then purified by distillation (Scheme 3a) [33]. The acid chloride was reacted with DL-homoserine lactone hydrobromide and pyridine as an acid scavenger at room temperature and then purified by flash column chromatography (Scheme 3b).

**Figure 1:**
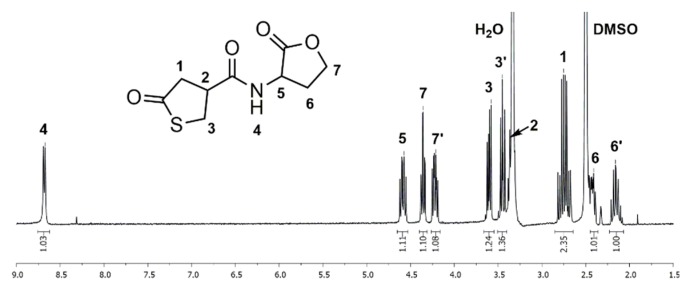
^1^H NMR spectrum of the thiolactone-lactone (**1**) measured in Deuterated dimethyl sulfoxide (DMSO-*d*_6_).

^1^H and ^13^C NMR spectra of thiolactone-homoserine lactone coupler **1** in DMSO-*d*_6_ show characteristic signals of the thiolactone and the lactone ring, proving the successful synthesis of the bifunctional coupler. In the ^1^H NMR spectrum (Figure 1), the distinctive signals for the thiolactone functionality are found as a multiplet at δ = 2.65–2.84 (Signal 1) ppm and two separate multiplets at δ = 3.31–3.40 and 3.41–3.51 (Signal 3) ppm for the two methylene protons contiguous to the sulfur atom. The signal of the methine group (Signal 2) adjacent to the carbonyl group is overlaid by the signal of residual water. The signals of the lactone moiety are shown as four multiplets at δ = 2.10–2.22, 2.38–2.46 (Signal 6), 4.17–4.26 and 4.31–4.39 (Signal 7) ppm for each proton of the methylene groups and a multiplet at δ = 4.54–4.64 (Signal 5) ppm for the methine group adjacent to the nitrogen atom. Additionally, the amide group exhibits a characteristic signal as a doublet at δ = 8.68 (Signal 4) ppm. In the ^13^C NMR spectrum (Figure S1) the thiolactone ring has specific signals at δ = 34.8 (Signal 4), 42.8 (Signal 3), 43.5 (Signal 2) and 206.5 (Signal 1) ppm. The signals of the lactone ring are found at δ = 28.1 (Signal 7), 48.2 (Signal 6), 65.3 (Signal 8) and 175.0 (Signal 9) ppm. The amide group shows a characteristic signal at δ = 170.7 (Signal 5) ppm. FTIR spectra (Figure S2), ESI mass spectrometry data (see experimental part), and elemental analysis (see experimental part) give further proof of the structure.

### 3.2. Model Reaction of Coupler **1** with Hexylamine

**Scheme 4:**
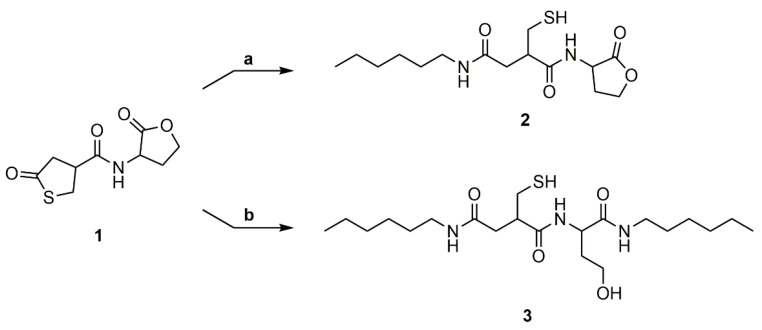
Model reactions to determine the regioselectivity of the amine addition with (**a**) 1 eq. hexylamine, deuterated dimethylformamide (DMF), rt and (**b**) 2 eq. hexylamine, DMF, 40 °C.

The reactivity of both cycles of the thiolactone-homoserine lactone coupler (**1**) was evaluated by model reactions with hexylamine (Scheme 4). The reaction trajectory was monitored by TLC and the conversion was verified by ^1^H NMR spectroscopy of the crude product. Reaction of one equivalent of hexylamine with **1** at room temperature gave compound **2**, in which the thiolactone ring is preferentially reacted (89% selectivity). The remaining 11% were a mixture of the disubstituted thiol-alcohol (**3**), the disulfides of **2** and **3**, and a non-identified side product. Reaction of coupler **1** with two equivalents of the amine at 40 °C resulted in full conversion of the reactants, however compound **3** was isolated with only a 34% yield. The low yield is attributed to strong interactions of the product with the stationary phase during the chromatographic purification step.

The ring-opening of the thiolactone shows specific signals in the ^1^H NMR spectrum (Figure S3). A multiplet at δ = 7.79–7.91 (Signal 8) ppm shows the amide group originating from the amine addition to the thiolactone ring. The signals of the methylene groups and the methine group are all shifted to a higher field at δ = 2.06–2.44 (Signal 1), 2.53–2.65 (Signal 3), and 2.75–2.85 (Signal 2) ppm, respectively. Ring-opening of the lactone also leads to a shift of the CH_2_ groups and the CH group of the lactone moiety to a higher field at δ = 1.54–1.72, 1.78–2.05 (Signal 6), 3.37–3.52 (Signal 7), and 4.14–4.24 (Signal 5) ppm (Figure S8). Furthermore, a signal at δ = 4.38–4.49 ppm exhibits the hydroxyl group emerging upon amine addition to the lactone. ^13^C NMR (Figures S4 and S9), H,H-COSY (Figures S5 and S10), HSQC (Figures S6 and S11) and NALDI-TOF (Figures S7 and S12, Tables S1 and S2) measurements give further proof of both structures.

### 3.3. Polyaddition Reaction with of PEG-Diamine to Coupler **1**

**Scheme 5:**
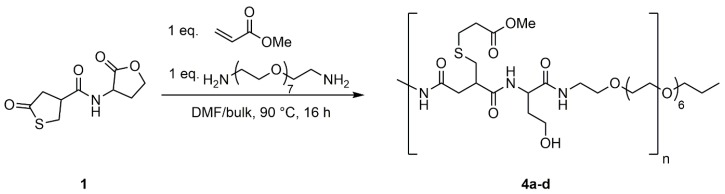
Polyaddition reaction of PEG-Diamine to thiolactone-lactone coupler (**1**) in the presence of methyl acrylate. Products were synthesized in various concentrations of **1** in DMF (**4a**: 0.5 M; **4b**: 1.0 M; **4c**: 1.5 M) and in bulk (**4d**).

To evaluate usability of the thiolactone-homoserine lactone coupler (**1**) as an AA-monomer in polyaddition reactions, **1** was reacted with PEG-diamine in DMF at various concentrations (0.5 M, 1.0 M, and 1.5 M) and in bulk (Scheme 5). The reaction was carried out at 90 °C to ensure full conversion of the coupler. Additionally, methyl acrylate was added to the reaction mixture to convert the thiol in situ to the respective thioether and suppress disulfide formation.

The ^1^H NMR spectrum shows the same shift to higher field of the thiolactone and the lactone ring upon ring-opening as for the model reactions (Figure S13). Additionally, signals for the added methyl acrylate can be found at δ = 2.97–3.10 ppm (Signal 5) for the methylene group adjacent to the thioether and δ = 3.58–3.72 ppm (Signal 8) for the methyl group of the acrylate. The methylene group next to the ester is overlaid by other signals of the opened thiolactone (δ = 2.06–2.74 ppm, Signals 2, 4, 6). Signals of the PEG-diamine are shown at δ = 3.28–3.43 ppm (Signal 13) and δ = 3.44–3.57 ppm (Signal 14), respectively.

**Figure 2:**
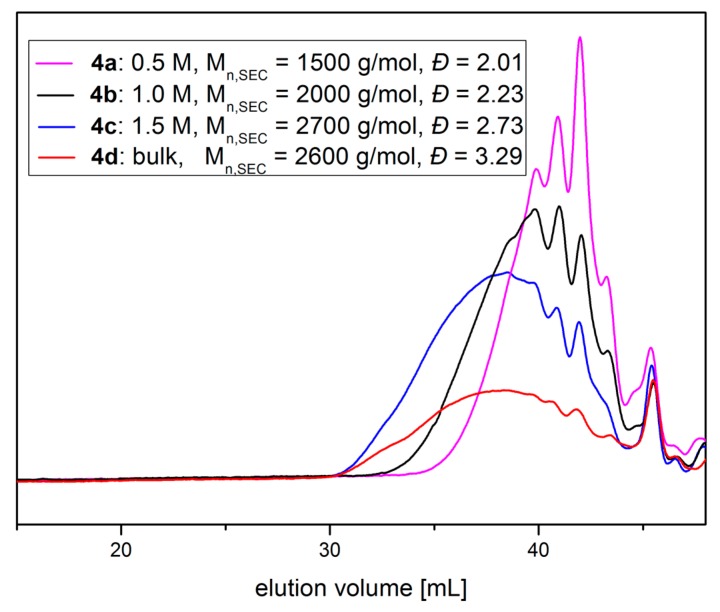
DMF-SEC traces of the reaction of thiolactone-lactone coupler **1** with PEG-diamine and methyl acrylate at c = 0.5 M (**4a**, magenta), 1.0 M (**4b**, black), 1.5 M (**4c**, blue) and in bulk (**4d**, red).

Formation of polymeric building blocks was evaluated by SEC analysis using DMF as eluent (Figure 2). An increase in concentration results in an increase of the molecular weight of the corresponding polymer. At 0.5 M mainly dimers are formed during the polyaddition reaction. This shifts to mainly tetramers at a concentration of 1.0 M and to higher oligomers at 1.5 M and in bulk. The signals appearing in the higher elution volume region can be assigned to oligomeric polyaddition products.

### 3.4. Functionalization of Polyglycidol (**5**) with DL-Homoserine Lactone Hydrobromide

Linear polyglycidol was prepared by anionic ring-opening polymerization of ethoxyethyl glycidyl ether with 3-phenyl-1-propanol as the initiator and removal of the acetal protecting groups under acidic conditions. [18] A polyglycidol with 26 repeating units (PG_26_) and *M*_n,SEC_ = 3100 g·mol^−1^ was obtained with a narrow molecular weight distribution (*Ð* = 1.14). The ^1^H, ^13^C NMR spectra and SEC analysis of PG_26_ (**5**) can be found in the supporting information (Figures S14–S16).

In preliminary experiments, it was observed that phenyl carbonates are excellent electrophiles for the substitution reaction with non-functionalized, primary amines. For the reaction with functionalized amines, such as homoserine lactone, the electrophilicity of the carbonate needs to be higher than the electrophilicity of the carbonyl group of the lactone ring. Phenyl carbonates are not electrophilic enough, leading to a reaction of the homoserine lactone with itself. In hydrolysis reactions, 4-nitrophenyl carbonates react faster by a factor of five, compared to phenyl carbonates [36]. We expected an equal increase in reactivity for the aminolysis reaction.

**Scheme 6:**
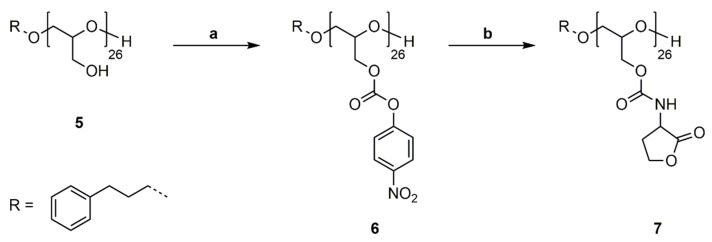
Synthetic pathway to Poly(Glycidyl Homoserine Lactonylcarbamate)_26_ (P(G^HSL^)_26_) (**7**). (**a**) Functionalization of PG_26_ (**5**) with 4-nitrophenyl chloroformate, pyridine/DCM, rt, 20 h; (**b**) Reaction of P(G^NPC^)_26_ (**6**) with DL-homoserine lactone hydrobromide, 4-DMAP, Et_3_N, DMF, rt, 20 h.

PG_26_ (**5**) was reacted with 4-nitrophenyl chloroformate in pyridine/DCM at room temperature (Scheme 6a). For purification poly(glycidyl-4-nitrophenylcarbonate)_26_ (**6**) was washed with water, 1M HCl solution (aq.) and saturated NaCl solution (aq.) to remove excess pyridine and pyridine hydrochloride and precipitated in methanol. The successful functionalization was confirmed by ^1^H, ^13^C NMR spectroscopy and SEC analysis (Figures S17–S19). P(G^NPC^)_26_ was subsequently reacted with DL-homoserine lactone hydrobromide in DMF at room temperature using catalytic amounts of 4-(dimethylamino)pyridine (4-DMAP) and Et_3_N as a base (Scheme 6b). The prepared poly(glycidyl homoserine lactonylcarbamate)_26_ (P(G^HSL^)_26_) (**7**) was purified by precipitation in methanol and characterized by ^1^H, ^13^C NMR spectroscopy and SEC analysis.

**Figure 3:**
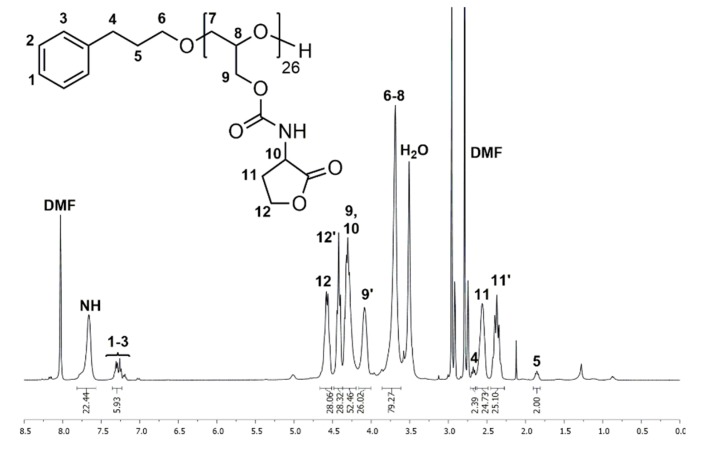
^1^H NMR spectrum of P(G^HSL^)_26_ (**7**) measured in DMF-*d*_7_.

^1^H and ^13^C NMR spectra of P(G^HSL^)_26_ (**7**) in DMF-*d*_7_ show characteristic signals of the homoserine lactonyl groups adjacent to the carbamate moieties, proving the successful functionalization of PG_26_. In the ^1^H NMR spectrum (Figure 3), the characteristic signals of the lactonyl functionality appear as four multiplets at δ = 2.27–2.45 and 2.49–2.63 (Signal 11) ppm for one methylene group and δ = 4.37–4.49 and 4.51–4.66 (Signal 12) ppm for the other methylene group. A multiplet at δ = 4.20–4.37 ppm (Signal 9, 10) shows the methylene group of the glycidol repeating unit and the single proton of the lactone ring adjacent to the carbamate moiety. In the ^13^C NMR spectrum (Figure S20), the distinctive signals of the homoserine lactone are found at δ = 28.7 (Signal 13), 50.3 (Signal 12) and 65.7 (Signal 14) ppm. Additionally, the specific signal of the carbamate groups is found at δ = 156.6 ppm (Signal 11).

The number of HSL groups attached to PG_26_ (**5**) was calculated by comparing the signal intensity of one methylene group of the 3-phenyl-1-propanol (Signal 5) used in the synthesis of **5** with signals 11 and 12. The absolute molecular weight (*M*_n,NMR_) was calculated likewise using the 3-phenyl-1-propyl end group as an internal reference (*M*_n,NMR_ = 5247 g·mol^−1^). Full functionalization of PG_26_ (**5**) was reached.

SEC analysis using DMF as the eluent confirms the synthesis of P(G^HSL^)_26_ (**7**) with *M*_n,SEC_ = 6900 g·mol^−1^ and a molecular weight distribution of *Ð* = 1.36 (Figure S21).

### 3.5. Ring-Opening of P(G^HSL^)_26_

**Scheme 7:**
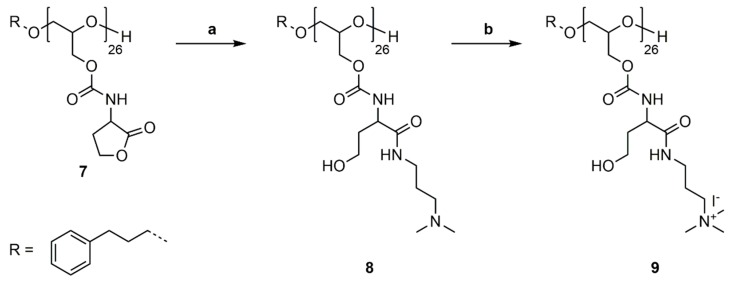
Synthetic pathway to P(G^HSL,o,q^)_26_ (**9**). (**a**) Addition of 3-(dimethylamino)-1-propylamine (DMAPA) to P(G^HSL^)_26_ (**7**), DMF, rt, 20 h; (**b**) Quaternization of P(G^HSL,o^)_26_ (**8**) with MeI, MeOH, reflux, 20 h.

P(G^HSL^)_26_ (**7**) was reacted with 3-(dimethylamino)-1-propylamine (DMAPA) and 4-DMAP as a nucleophilic catalyst in a ring-opening reaction in DMF at room temperature (Scheme 7a). The synthesized P(G^HSL,o^)_26_ (**8**) was purified by precipitation in diethyl ether, followed by dialysis in MeOH and characterized by ^1^H, ^13^C NMR spectroscopy and SEC analysis (Figures S22–S24).

^1^H and ^13^C NMR spectra in DMSO-*d*_6_ exhibit characteristic signals for the DMAPA group and the opened lactone. In the ^1^H NMR spectrum the distinctive signals of the DMAPA functionality appear as two multiplets at δ = 1.41–1.58 ppm (Signal 14) and δ = 3.03–3.10 ppm (Signal 13), and a multiplet at δ = 2.18 ppm (Signal 15). The singlet at δ = 2.09 ppm (Signal 16) represents the methyl groups vicinal to the amine. The characteristic signals of the opened lactone appear as two multiplets at 1.66–1.77 ppm (Signal 11) and 3.81–4.25 ppm (Signal 10). The second multiplet also contains the methylene groups of the polyglycidol repeating unit adjacent to the carbamate. In the ^13^C NMR spectrum the distinctive signals appear at δ = 26.9 (Signal 17), 37.2 (Signal 16), 45.2 (Signal 19), and 57.7 (Signal 18) ppm for the DMAPA group and at δ = 35.1 (Signal 13), 52.2 (Signal 12), and 56.8 (Signal 14) ppm for the opened lactone. The number of DMAPA moieties attached to PG_26_ was calculated as described previously.

SEC analysis using DMF as the eluent confirms the successful ring-opening of P(G^HSL^)_26_ (**7**) with *M*_n,SEC_ = 8900 g·mol^−1^ and a molecular weight distribution of *Ð* = 1.32.

### 3.6. Quaternization of P(G^HSL,o^)_26_

As a model reaction for further functionalization, the quaternization of P(G^HSL,o^)_26_ (**8**) was conducted with methyl iodide in methanol under reflux (Scheme 7b). The solvent and excess methyl iodide was removed under reduced pressure and P(G^HSL,o,q^)_26_ (**9**) characterized by ^1^H and ^13^C NMR spectroscopy (Figures S25 and S26).

^1^H and ^13^C NMR spectra in D_2_O show distinctive signals of the quaternary amine functionality. In the ^1^H NMR, the singlet at δ = 3.18 ppm (Signal 16) represents the methyl groups vicinal to the amine and the multiplet at δ = 3.25–3.47 ppm (Signal 15) shows the methylene groups adjacent to the amine and the carbamate. The third methylene group appears at δ = 1.95–2.08 (Signal 14) ppm. In the ^13^C NMR, spectrum the characteristic signals of the quaternary amine moiety appear at δ = 22.8 (Signal 17), 36.2 (Signal 16), 53.2 (Signal 19), and 64.2 (Signal 18) ppm. Both spectra confirm the successful quaternization of P(G^HSL,o^)_26_ (**8**).

## 4. Conclusions

We have developed two synthetic strategies using homoserine lactone for the preparation of polymers. The first strategy comprises the synthesis of a bifunctional thiolactone-lactone coupler. The regioselectivity of amine addition towards the thiolactone functionality was shown in model reactions with hexylamine at room temperature. At an elevated temperature, an amine reacted with both heterocycles. The principle of these model reactions was used to prepare functional polyamide oligomers by polyaddition reaction with a PEG-diamine (*M*_n_ = 368.74 g·mol^−1^).

The second strategy includes the functionalization of linear polyglycidol with homoserine lactone. The successful synthesis was confirmed by ^1^H, ^13^C NMR spectroscopy, and SEC analysis. Homoserine lactone groups were subsequently opened by addition of 3-(dimethylamino)-1-propylamine and the tertiary amine was quaternized with methyl iodide.

Future work will further exploit the possibility of the introduction of multifunctionality into the polymers both synthetic strategies offer.

## Supplementary Materials

The following are available online at www.mdpi.com/2073-4360/9/4/130/s1, Figure S1: ^13^C NMR spectrum of thiolactone-lactone (**1**) measured in DMSO-*d*_6_, Figure S2: FTIR spectrum of thiolactone-lactone (**1**), Figure S3: ^1^H NMR spectrum of compound **2** measured in DMSO-*d*_6_, Figure S4: ^13^C NMR spectrum of compound **2** measured in DMSO-*d*_6_, Figure S5: H,H-COSY NMR spectrum of compound **2** measured in DMSO-*d*_6_, Figure S6: HSQC NMR spectrum of compound **2** measured in DMSO-*d*_6_, Figure S7: NALDI-TOF spectrum of compound **2**, Table S1: Identified signals of the NALDI-TOF measurement of the addition product of the addition of one equivalent hexylamine to the thiolactone-lactone coupler (**1**), Figure S8: ^1^H NMR spectrum of compound **3** measured in DMSO-*d*_6_, Figure S9: ^13^C NMR spectrum of compound **3** measured in DMSO-*d*_6_, Figure S10: H,H-COSY NMR spectrum of compound **3** measured in DMSO-*d*_6_, Figure S11: HSQC NMR spectrum of compound **3** measured in DMSO-*d*_6_, Figure S12: NALDI-TOF spectrum of compound **3**, Table S2: Identified signals of the NALDI-TOF measurement of the addition product of the addition of two equivalents hexylamine to the thiolactone-lactone coupler (**1**), Table S3: Reagent ratios for the synthesis of **4a**–**d** (*T* = 90 °C, *t* = 16 h), Figure S13: ^1^H NMR spectrum of the reaction of thiolactone-lactone (**4**) with PEG-diamine measured in DMSO-*d*_6_, Figure S14: ^1^H NMR spectrum of PG_26_ (**5**) measured in DMSO-*d*_6_, Figure S15: ^13^C NMR spectrum of PG_26_ (**5**) measured in DMSO-*d*_6_, Figure S16: DMF-SEC traces of PG_26_ (**5**), Figure S17: ^1^H NMR spectrum of P(G^NPC^)_26_ (**6**) measured in DMSO-*d*_6_, Figure S18: ^13^C NMR spectrum of P(G^NPC^)_26_ (**6**) measured in DMSO-*d*_6_, Figure S19: DMF-SEC traces of P(G^NPC^)_26_ (**6**), Figure S20: ^13^C NMR spectrum of P(G^HSL^)_26_ (**7**) measured in DMF-*d*_7_, Figure S21: DMF-SEC traces of P(G^HSL^)_26_ (**7**), Figure S22: ^1^H NMR spectrum of P(G^HSL,o^)_26_ (**8**) measured in DMSO-*d*_6_, Figure S23: ^13^C NMR spectrum of P(G^HSL,o^)_26_ (**8**) measured in DMSO-*d*_6_, Figure S24: DMF-SEC traces of P(G^HSL,o^)_26_ (**8**), Figure S25: ^1^H NMR spectrum of P(G^HSL,o,q^)_26_ (**9**) measured in D_2_O, Figure S26: ^13^C NMR spectrum of P(G^HSL,o,q^)_26_ (**9**) measured in D_2_O.
