# Supplementary material for: Homoserine Lactone as a Structural Key Element for the Synthesis of Multifunctional Polymers
Source: Polymers (Basel). 2017 Apr 5;9(4):130. doi: 10.3390/polym9040130 (PMC6432242; doi:10.3390/polym9040130)
Supplement: Supplementary file 1 [file polymers-09-00130-s001.pdf]

polymers

Supporting Information

Homoserine lactone as a structural key element for the synthesis of multifunctional polymers

Fabian Marquardt, Stefan Mommer, Justin Lange, Pascal M. Jeschenko, Helmut Keul*, Martin Möller*

Institute of Technical and Macromolecular Chemistry, RWTH Aachen University and DWI-Leibniz-Institute for Interactive Materials, Forckenbeckstr. 50, D-52056 Aachen, Germany; Marquardt@dwi.rwth-aachen.de (F. M.); Mommer@dwi.rwth-aachen.de (S. M.); Justin.lange@rwth-aachen.de (J. L.); Pascal.Jeschenko@rwth-aachen.de (P. M. J.)

* Correspondence: Keul@dwi.rwth-aachen.de (H. K.); Tel.: +49-241-80-26438; Moeller@dwi.rwth-aachen.de (M. M.); Tel.: +49-241-80-23302

1. Synthesis of the thiolactone-lactone coupler (1)

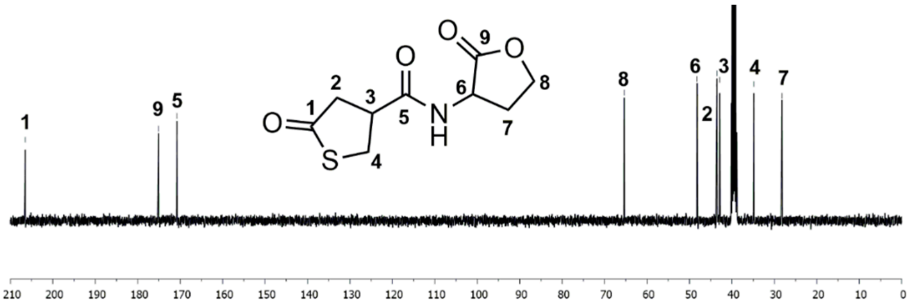

Figure S1. ^{13}C NMR spectrum of thiolactone-lactone (**1**) measured in DMSO-d_6.

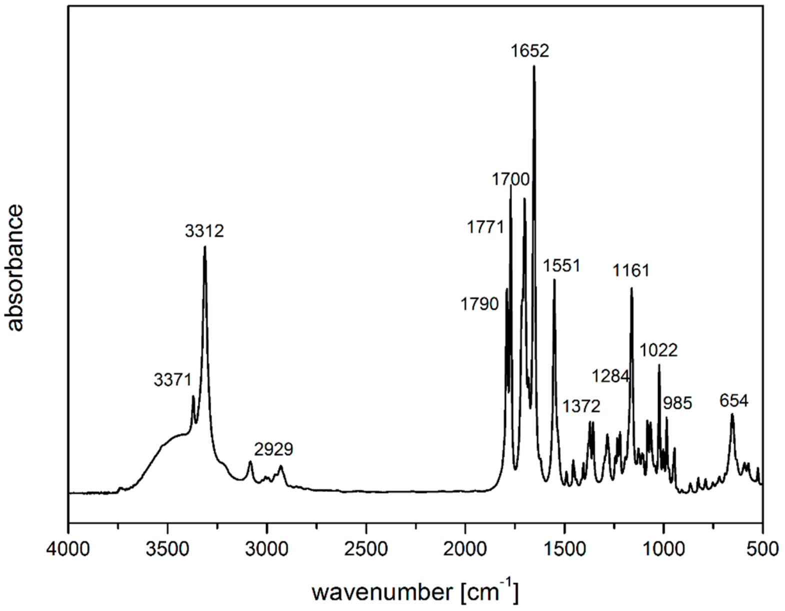

Figure S2. FTIR spectrum of thiolactone-lactone (**1**).

2. Model reaction of coupler 1 with hexylamine in a 1:1 molar ratio

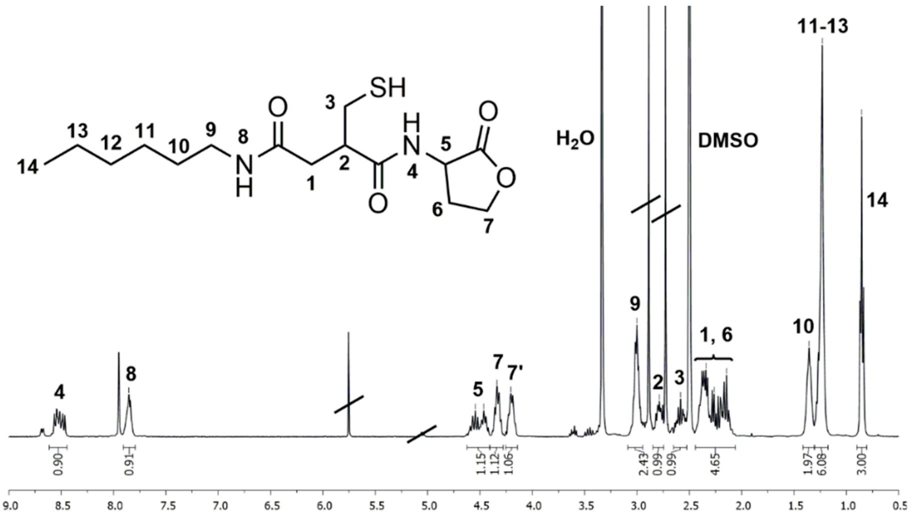

Figure S3. ^{1}H NMR spectrum of compound **2** measured in DMSO-d_6.

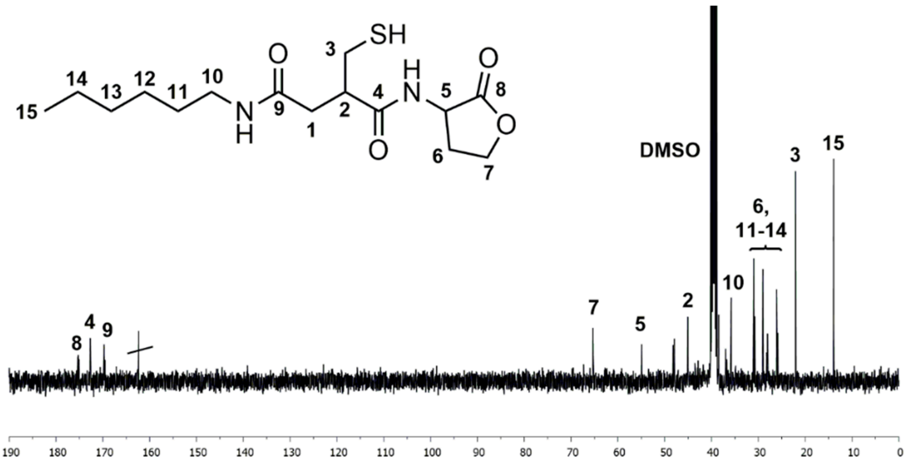

Figure S4. ^{13}C NMR spectrum of compound **2** measured in DMSO-d_6.

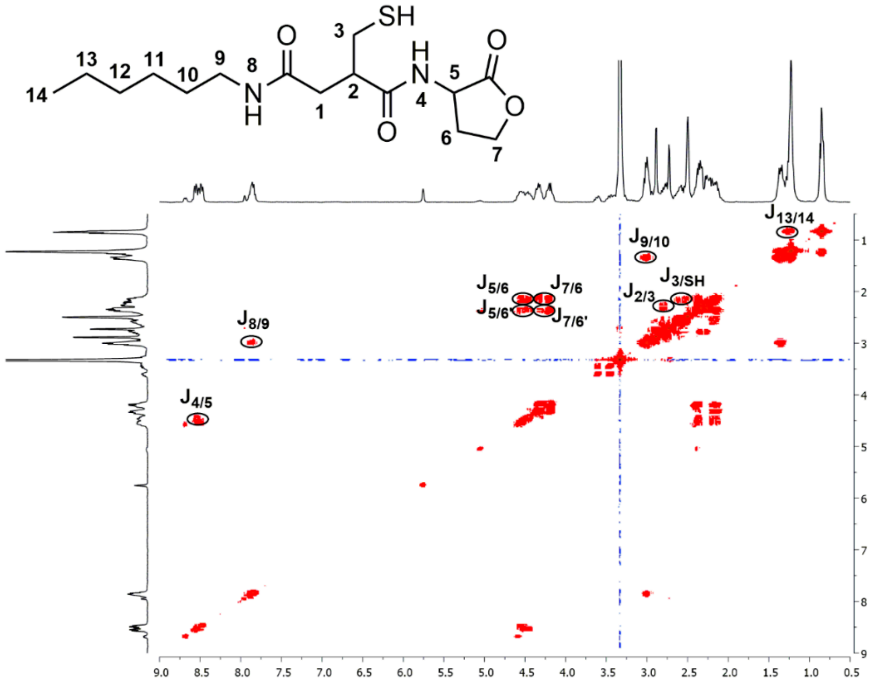

Figure S5. H,H-COSY NMR spectrum of compound **2** measured in DMSO-d_6.

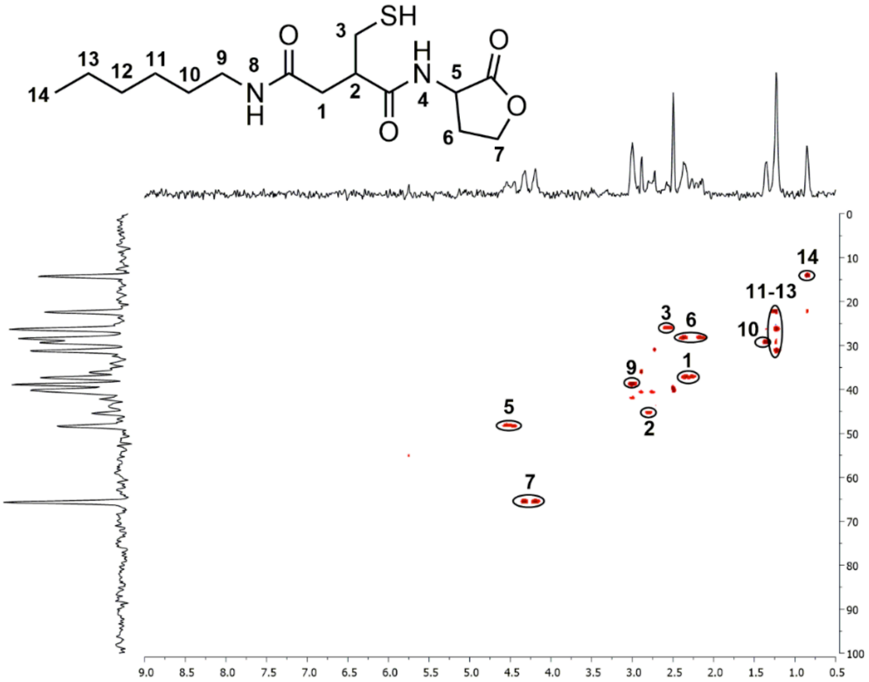

Figure S6. HSQC NMR spectrum of compound **2** measured in DMSO-d_6.

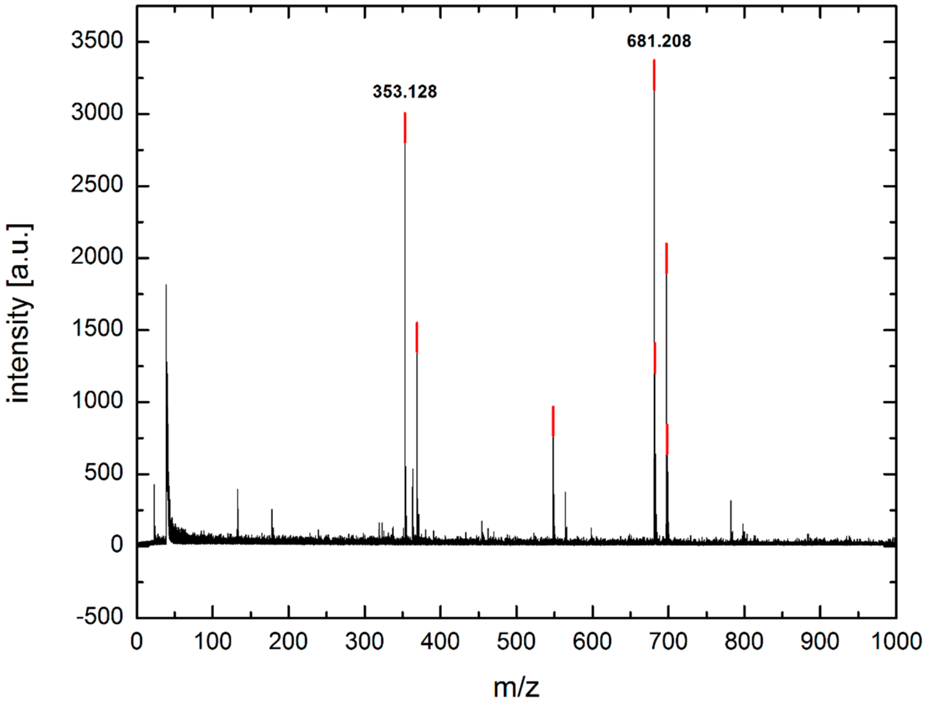

Figure S7. NALDI-TOF spectrum of compound **2**.

Table S1. Identified signals of the NALDI-TOF measurement of the addition product of the addition one equivalent hexylamine to the thiolactone-lactone coupler (**1**). Reported signals describe masses of [M + Na^+] (MW = 22.99 g · mol^{-1}).

Product	m/z (calculated)	m/z (found)	intensity [a.u.]
**2**	353.15	353.128	2902
Disulfide of **2**	681.29	681.208	3270

3. Model reaction of coupler 1 with hexylamine in a 1:2 molar ratio

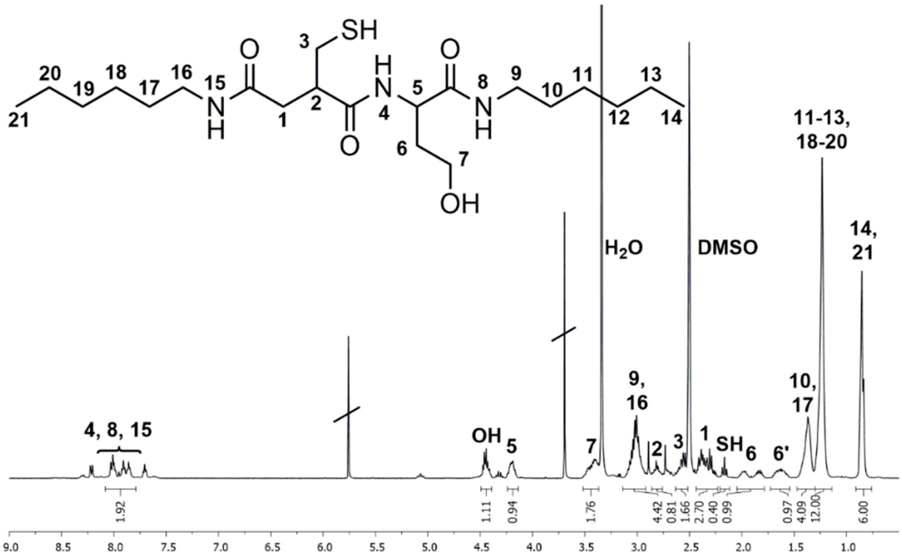

Figure S8. ^{1}H NMR spectrum of compound **3** measured in DMSO-d_6.

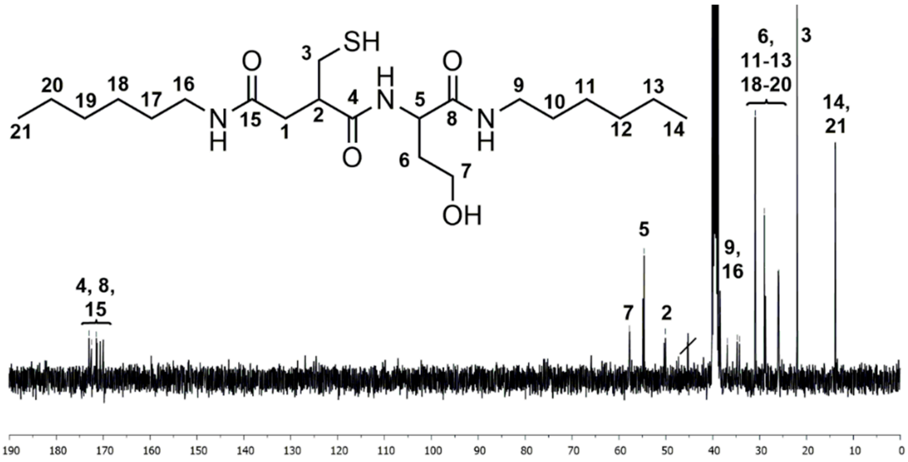

Figure S9. ^{13}C NMR spectrum of compound **3** measured in DMSO-d_6.

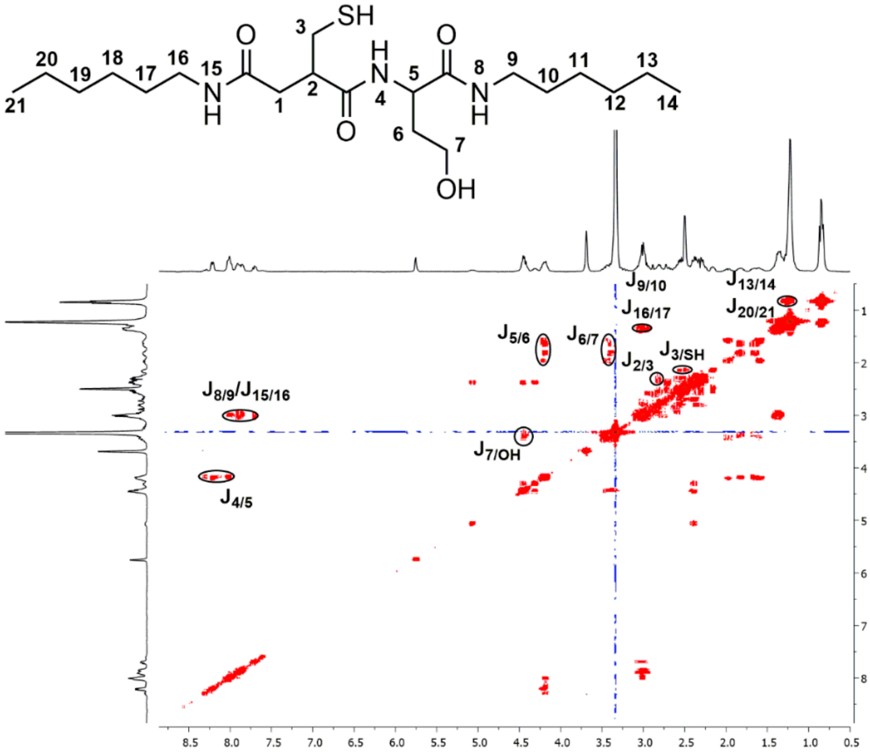

Figure S10. H,H-COSY NMR spectrum of compound **3** measured in DMSO-d_6.

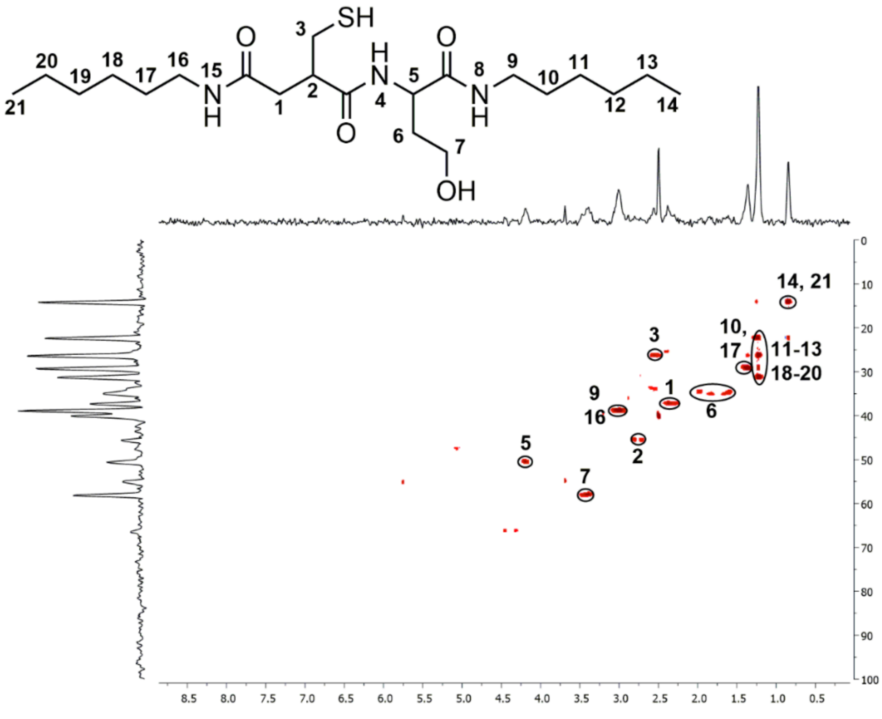

Figure S11. HSQC NMR spectrum of compound **3** measured in DMSO-d_6.

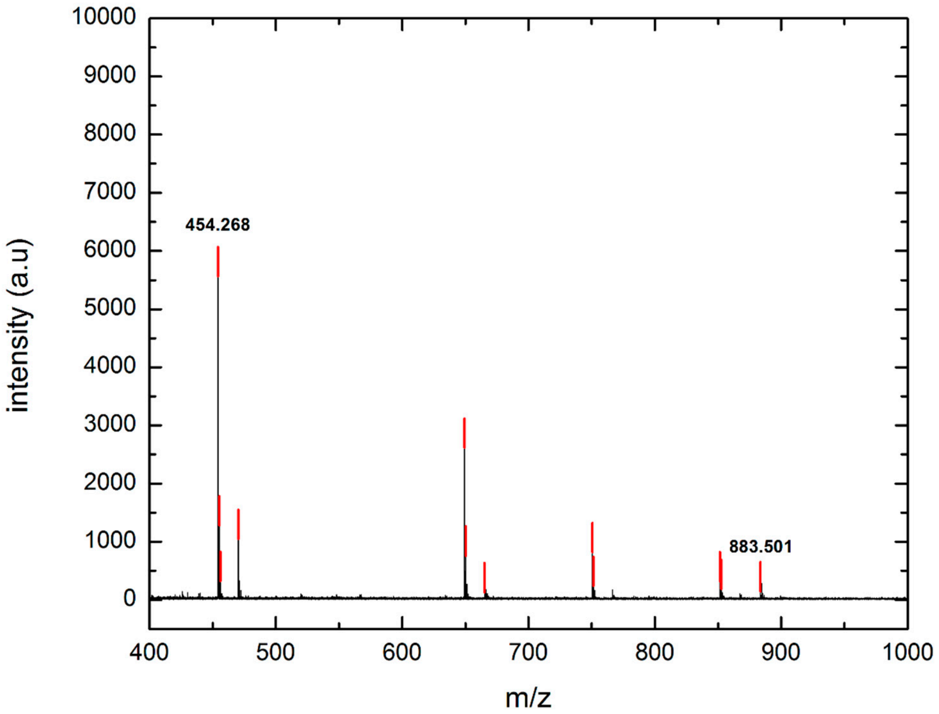

Figure S12. NALDI-TOF spectrum of compound **3**.

Table S2. Identified signals of the NALDI-TOF measurement of the addition product of the addition of two equivalents hexylamine to the thiolactone-lactone coupler (**1**). Reported signals describe masses of [M + Na+] (MW = 22.99 g · mol-1).

Product	m/z (calculated)	m/z (found)	intensity [a.u.]
**3**	454.27	454.268	5819
Disulfide of **3**	883.53	883.501	403

4. Polyaddition reaction with of PEG-diamine to coupler 1

Table S3. Reagent ratios for the synthesis of **4a-d** (T = 90 °C, t = 16 h).

polymer	m_1 [g]	m_{MA} [g]	$m_{PEG\text{-}diamine}$ [g]	V_{DMF} [mL]	c [g · mol^{-1}]
**7a**	0.249	0.094	0.400	2.170	0.5
**7b**	0.100	0.038	0.161	0.436	1.0
**7c**	0.100	0.038	0.161	0.291	1.5
**7d**	0.100	0.038	0.161	-	-

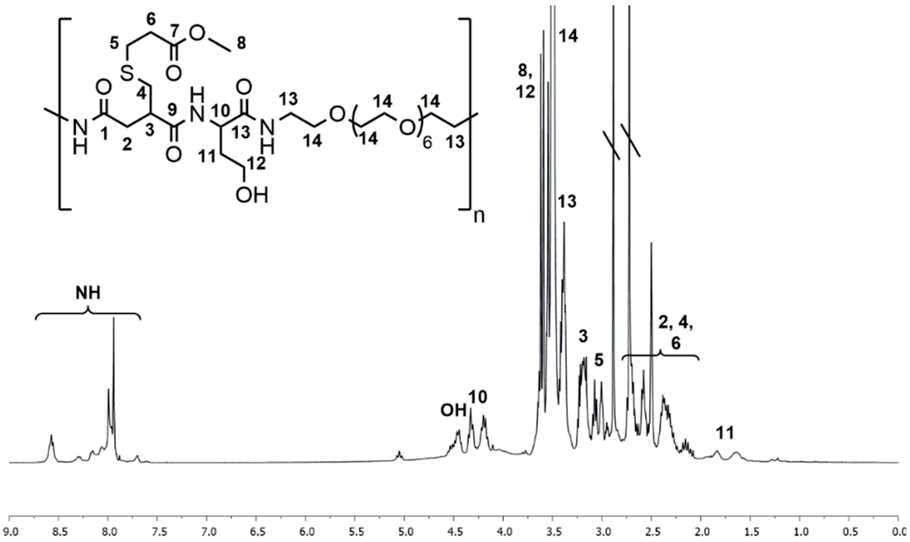

Figure S13. ^{1}H NMR spectrum of the reaction of thiolactone-lactone (**4**) with PEG-diamine measured in DMSO-d_6.

5. Synthesis of PG$_{26}$ (5)

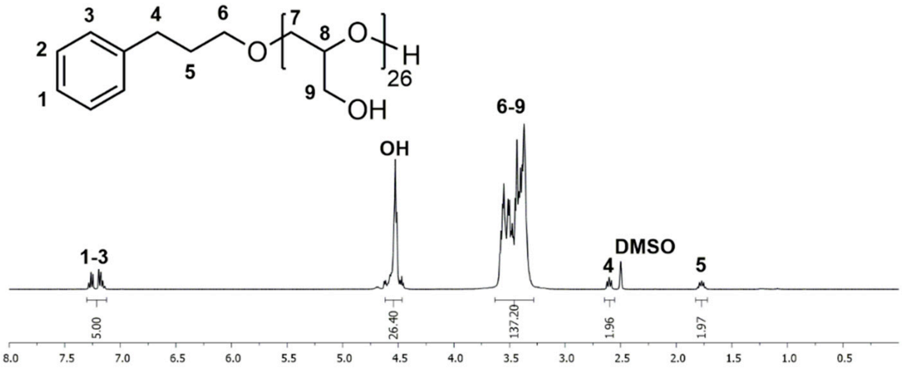

Figure S14. ^{1}H NMR spectrum of PG$_{26}$ (**5**) measured in DMSO-d_6.

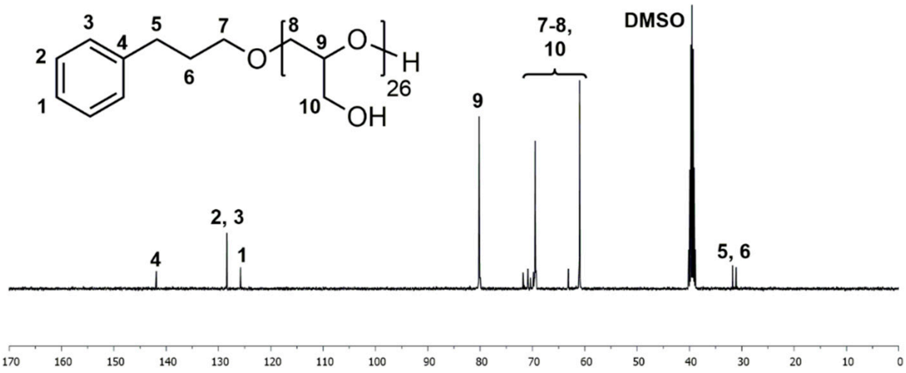

Figure S15. ^{13}C NMR spectrum of PG$_{26}$ (**5**) measured in DMSO-d_6.

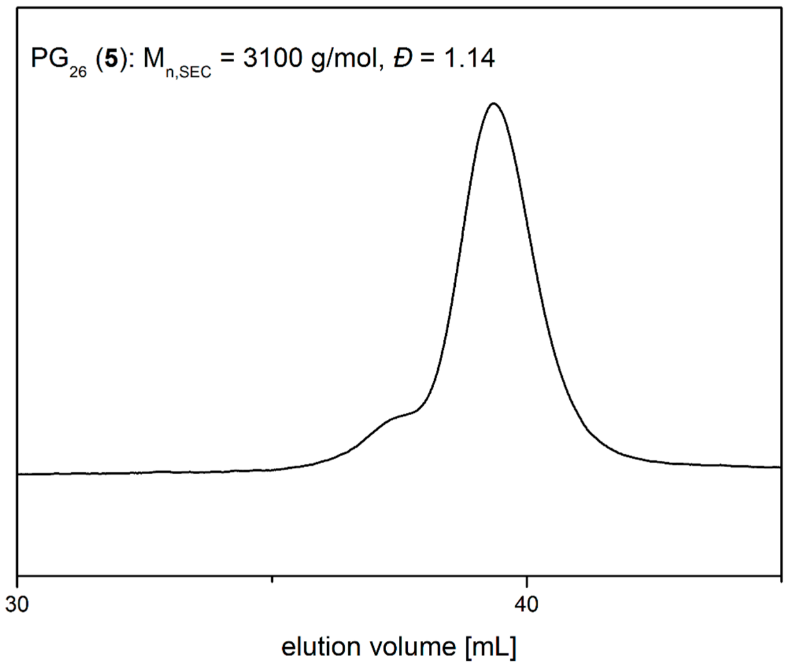

Figure S16. DMF-SEC traces of PG_{26} (**5**).

6. Functionalization of polyglycidol (5) with DL-homoserine lactone hydrobromide

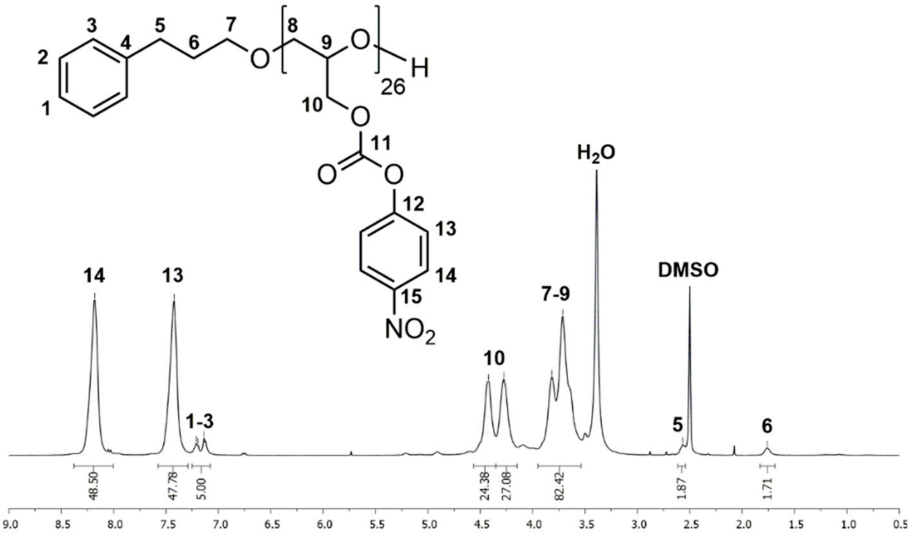

Figure S17. ^{1}H NMR spectrum of $P(G^{NPC})_{26}$ (**6**) measured in DMSO-d_6.

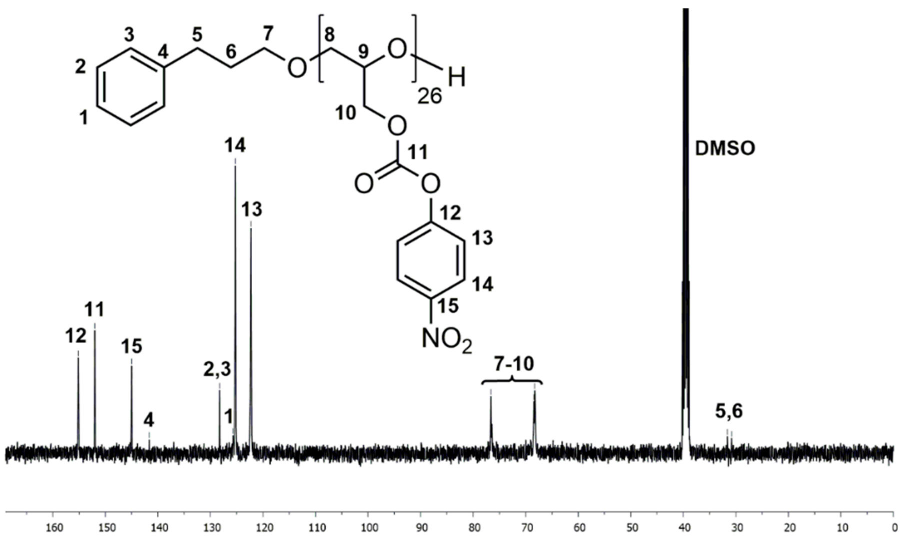

Figure S18. ^{13}C NMR spectrum of P(G^{NPC})$_{26}$ (**6**) measured in DMSO-d_6.

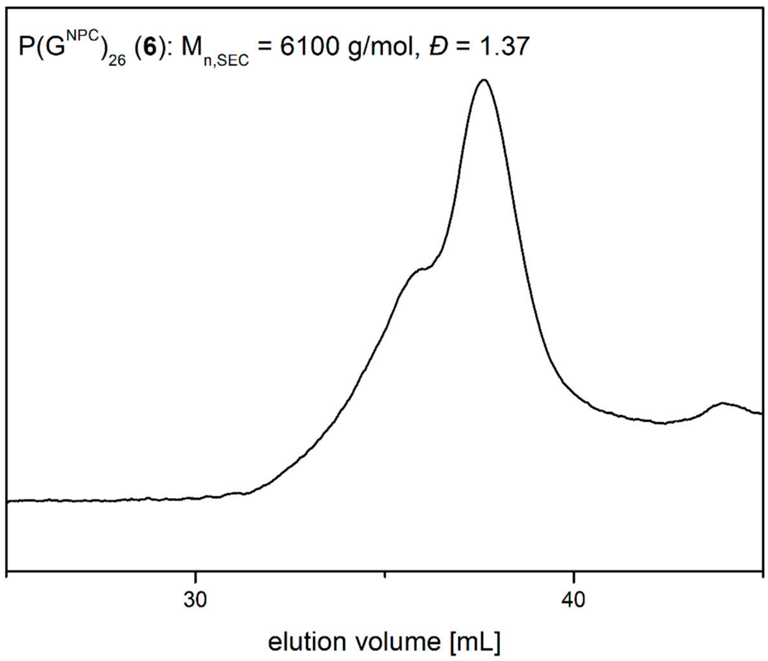

Figure S19. DMF-SEC traces of P(G^{NPC})$_{26}$ (**6**).

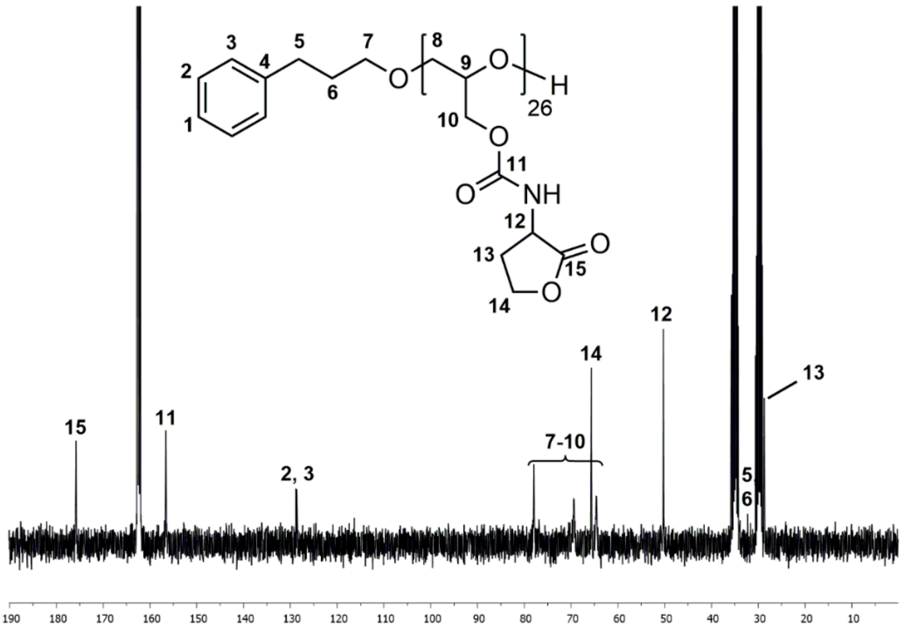

Figure S20. ^{13}C NMR spectrum of P(G^{HSL})$_{26}$ (**7**) measured in DMF-*d*$_7$.

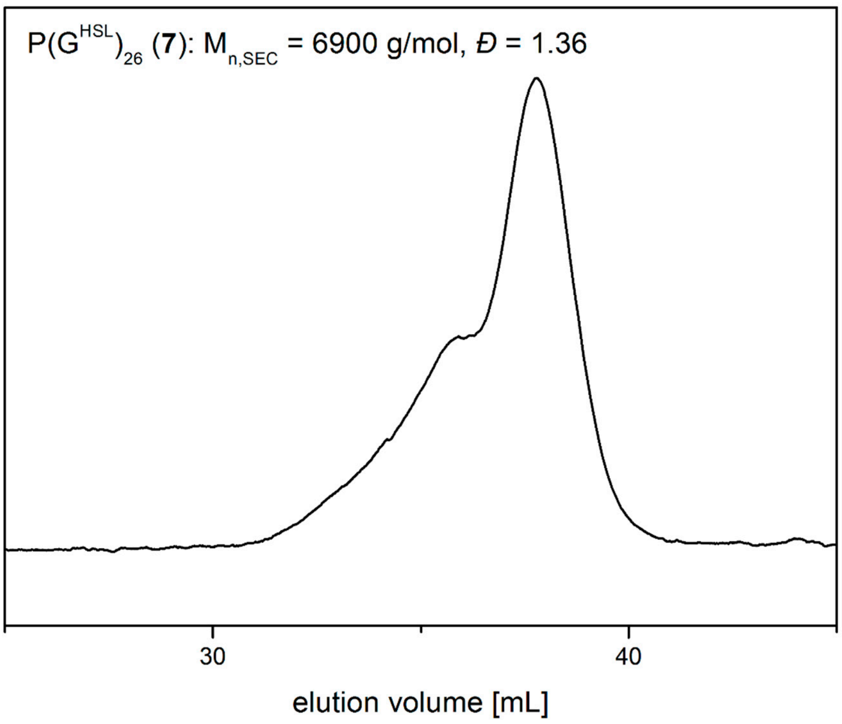

Figure S21. DMF-SEC traces of P(G^{HSL})$_{26}$ (**7**).

7. Ring-opening of P(G^{HSL})$_{26}$

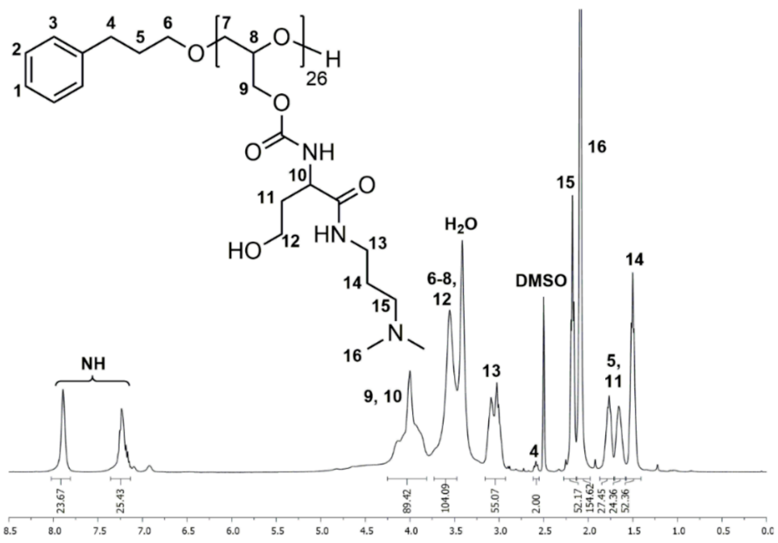

Figure S22. ^{1}H NMR spectrum of P($G^{HSL,o}$)$_{26}$ (**8**) measured in DMSO-d_6.

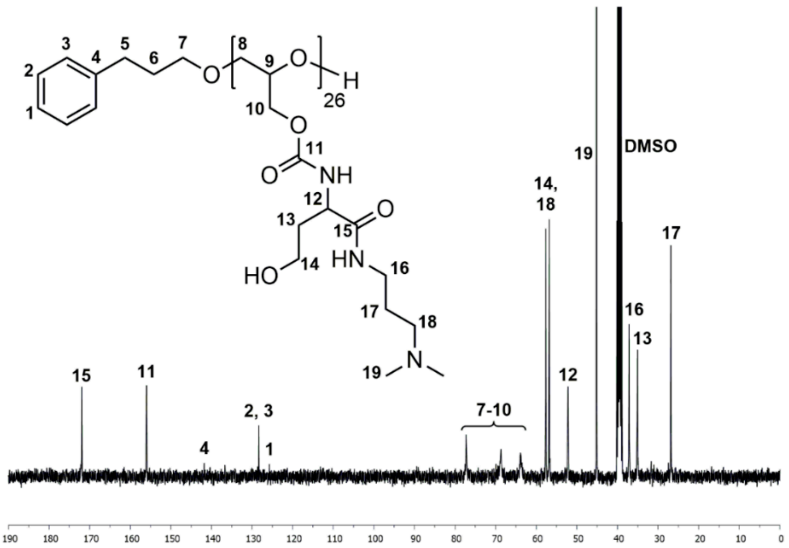

Figure S23. ^{13}C NMR spectrum of P($G^{HSL,o}$)$_{26}$ (**8**) measured in DMSO-d_6.

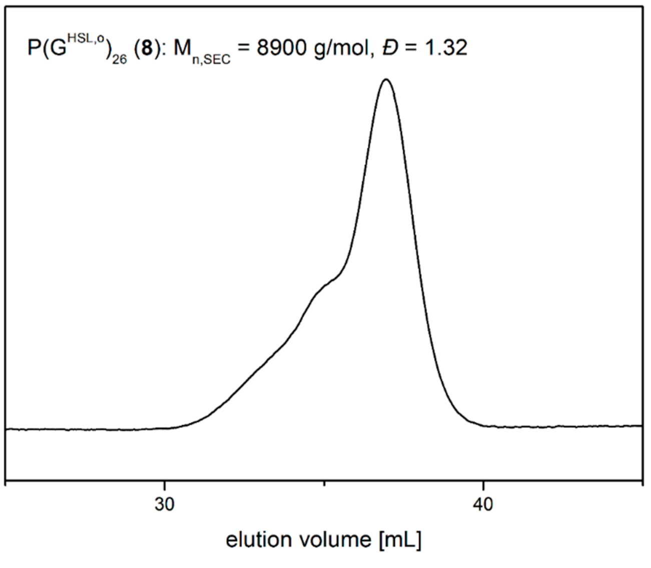

Figure S24. DMF-SEC traces of P($G^{HSL,o}$)$_{26}$ (**8**).

8. Quaternization of P(G^{HSL,o})$_{26}$

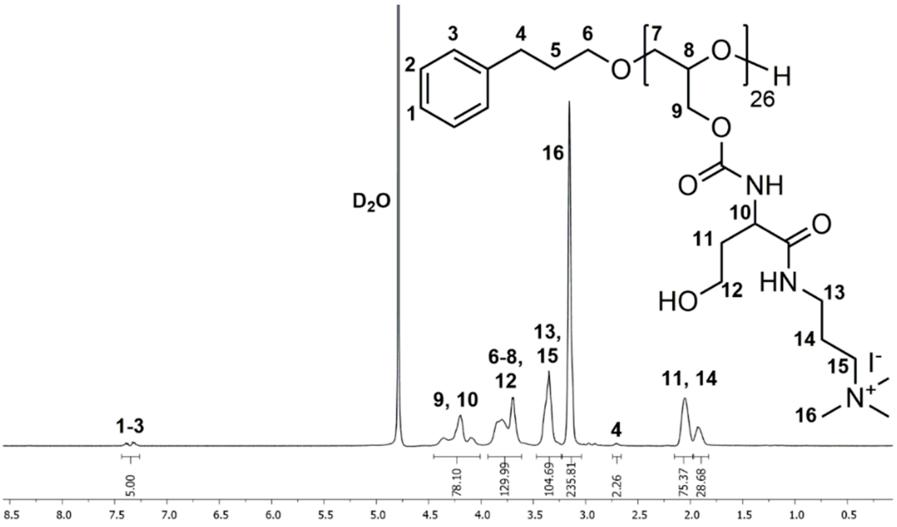

Figure S25. ^{1}H NMR spectrum of P(G^{HSL,o,q})$_{26}$ (**9**) measured in D_2O.

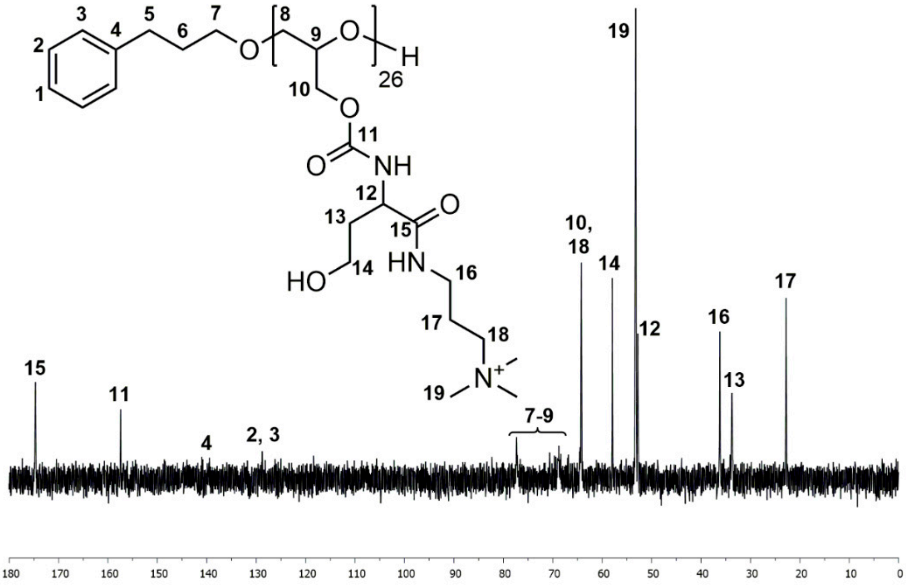

Figure S26. ^{13}C NMR spectrum of P(G^{HSL,o,q})$_{26}$ (**9**) measured in D_2O.
